# Kinetic and thermodynamic analysis defines roles for two metal ions in DNA polymerase specificity and catalysis

**DOI:** 10.1074/jbc.RA120.016489

**Published:** 2020-12-17

**Authors:** Shanzhong Gong, Serdal Kirmizialtin, Adrienne Chang, Joshua E. Mayfield, Yan Jessie Zhang, Kenneth A. Johnson

**Affiliations:** 1 Department of Molecular Biosciences, The University of Texas at Austin, Austin, Texas, USA; 2 Chemistry Program, Science Division, New York University Abu Dhabi, Abu Dhabi, United Arab Emirates

**Keywords:** DNA replication, magnesium, molecular dynamics, conformational change, enzyme catalysis, transient kinetics, induced fit, enzyme specificity, metal ions, dNTP, deoxynucleoside triphosphate, dTTP, thymidine triphosphate, ED, enzyme–DNA complex, ED_dd_, enzyme–DNA complex with a dideoxy-terminated primer strand, HIV-RT, human immunodeficiency virus reverse transcriptase, MD, molecular dynamics, MDCC, 7-diethylamino-3-((((2-maleimidyl)ethyl)amino)carbonyl) coumarin, Mg_A_, catalytic Mg^2+^, Mg_B_, nucleotide-bound Mg^2+^

## Abstract

Magnesium ions play a critical role in catalysis by many enzymes and contribute to the fidelity of DNA polymerases through a two-metal ion mechanism. However, specificity is a kinetic phenomenon and the roles of Mg^2+^ ions in each step in the catalysis have not been resolved. We first examined the roles of Mg^2+^ by kinetic analysis of single nucleotide incorporation catalyzed by HIV reverse transcriptase. We show that Mg.dNTP binding induces an enzyme conformational change at a rate that is independent of free Mg^2+^ concentration. Subsequently, the second Mg^2+^ binds to the closed state of the enzyme–DNA–Mg.dNTP complex (*K*_*d*_ = 3.7 mM) to facilitate catalysis. Weak binding of the catalytic Mg^2+^ contributes to fidelity by sampling the correctly aligned substrate without perturbing the equilibrium for nucleotide binding at physiological Mg^2+^ concentrations. An increase of the Mg^2+^ concentration from 0.25 to 10 mM increases nucleotide specificity (*k*_cat_/*K*_*m*_) 12-fold largely by increasing the rate of the chemistry relative to the rate of nucleotide release. Mg^2+^ binds very weakly (*K*_*d*_ ≤ 37 mM) to the open state of the enzyme. Analysis of published crystal structures showed that HIV reverse transcriptase binds only two metal ions prior to incorporation of a correct base pair. Molecular dynamics simulations support the two-metal ion mechanism and the kinetic data indicating weak binding of the catalytic Mg^2+^. Molecular dynamics simulations also revealed the importance of the divalent cation cloud surrounding exposed phosphates on the DNA. These results enlighten the roles of the two metal ions in the specificity of DNA polymerases.

Metal ions play critical roles in many biological activities including DNA replication, DNA repair, and transcription as well as other phosphoryl-group transfer reactions, including some ribozymes, adenylyl cyclase, and protein kinases (1, 2, 3, 4, 5). They stabilize the structures of proteins and nucleic acids and promote the catalytic activities (6). Magnesium ion (Mg^2+^) serves as the primary metal ion for catalysis, owing to its natural abundance *in vivo* and restricted coordination geometry conferring high stereoselectivity (7). The role of metal ions in DNA polymerization and hydrolysis was first described by Steitz in 1993 (1) who proposed a two-metal-ion mechanism in which one metal ion forms a tight complex with the incoming nucleotide by coordinating with nonbridging oxygens from all three phosphates (8, 9). A second metal ion reduces the pK_a_ of the 3'-OH group for polymerization (or of a water molecule for hydrolysis), thereby activating the nucleophile and bringing it close to the α-phosphate at the reaction center. The coordinated action of two metal ions, water molecules, and several surrounding acidic residues helps to stabilize the transition state (6, 10). After polymerization, Mg-pyrophosphate (Mg.PPi) is released from the enzyme (1, 11). The two-metal-ion mechanism is supported by many crystal structures of DNA polymerases (2). However, crystal structures only provide a static picture of the active site, do not reveal weakly bound metal ions or their dynamic movements, and do not reveal the pathway or thermodynamics of the reaction. Moreover, dideoxy-terminated primer, calcium ions, or nonhydrolyzable nucleotide analogs are usually used in crystal structures to prevent catalysis and these may disrupt the active site geometry and the conformational state of the enzyme. Recently it has been proposed that a third metal ion may be required for catalysis (12, 13). The third metal ion is seen predominantly in DNA repair enzymes and may be associated with the stabilization of the product pyrophosphate (PPi) (14), but the role of a possible third metal ion in catalysis remains unresolved (15) and has not been seen in higher-fidelity enzymes.

Enzyme mechanism and specificity are kinetic phenomena that cannot be addressed by structural studies alone. Rather, structural studies provide the framework to design and interpret kinetic and mechanistic experiments, so the two approaches together provide new insights. To further understand the role of Mg^2+^ in catalysis and specificity, studies on the dynamics of the metal ions under biologically relevant conditions are required. HIV reverse transcriptase (HIV-RT) belongs to the A family of moderate- to high-fidelity enzymes. It serves as a good candidate for studying the two-metal-ion mechanism because kinetic characterization of single nucleotide incorporation has established the mechanistic basis for polymerase fidelity and has defined the role of a nucleotide-induced conformational change step in specificity (16, 17). These studies have established the following minimal pathway for nucleotide incorporation for HIV-RT.

Specificity for cognate nucleotide incorporation by HIV-RT is a function on an induced-fit mechanism where *k*_cat_*/K*_*m*_ is defined by the rate of the fast conformational change to the closed state (*k*_*2*_) divided by the *K*_*d*_ for the weak binding of nucleotide to the open state of the enzyme (18, 19) as shown in Figure 1 (*k*_cat_*/K*_*m*_ *= K*_*1*_*k*_*2*_). The closed state traps the nucleotide and aligns catalytic residues to facilitate fast catalysis. Under the conditions used in this study, the release of Mg.PP_i_ is fast, so the data collected for the forward reaction cannot define *k*_*−3*_, the reverse of chemistry. With an RNA template, release of Mg.PPi is slow, so the chemical reaction approaches an equilibrium to provide an estimate of *k*_*−3*_ *based* on the concentration dependence of the amplitude of the reaction (20).

**Figure 1:**
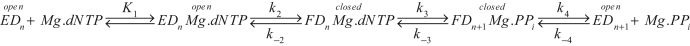
**Pathway of DNA polymerization.** The minimal reaction pathway is shown where *ED*_*n*_ and *FD*_*n*_ represent the enzyme–DNA complex in the *open* and *closed* states, respectively, as observed in crystal structures and shown to be kinetically important (18).

Although it is clear that Mg.dNTP is the substrate for the reaction, the roles of the second Mg^2+^ in each of these steps are not known, and without this information the role of Mg^2+^ in specificity cannot be established. For example, what is the order of binding the second Mg^2+^ relative to other steps in the pathway? Does Mg^2+^ bind to the open state? What is the net *K*_*d*_ for binding the catalytic Mg^2+^ to either the open or the closed state? How does free Mg^2+^ ion affect ground-state binding, conformational change, chemistry, and PPi release? How does the free Mg^2+^ concentration alter nucleotide specificity? Is there evidence for the involvement of a third metal ion? In this study, we addressed these questions by examining the Mg^2+^ concentration dependence of each step in the reaction pathway in order to estimate the initial binding affinity of Mg.dNTP to the open state of the enzyme, the rate of the nucleotide-induced conformational change, the rate of nucleotide release before chemistry, the rate of the chemical reaction, and the rate of product release. This analysis allows us to resolve the contributions of each metal ion toward enzyme specificity. We also use molecular dynamics (MD) simulations to view the binding of Mg^2+^ to multiple sites on the enzyme–DNA complex. The results from MD simulations are consistent with experimental measurements of binding affinity and provide molecular details for aspects of Mg^2+^ binding that cannot be observed directly.

The studies performed here provide new insights toward understanding the role of metal ions in DNA polymerase fidelity. We show that Mg.dNTP is necessary and sufficient to induce the conformational change from the *open* to the *closed* state. The second Mg^2+^ binds after the conformational change, stabilizes the closed state, and stimulates the chemical reaction. Accordingly, we will refer the second metal ion as the *catalytic Mg*^*2+*^ to distinguish it from the *nucleotide-bound* Mg^2+^, although it must be clear that both metal ions are required for catalysis. In the course of performing these experiments, we also developed a simplified method to accurately define concentrations of free Mg^2+^ and Mg.dNTP in solution using a Mg-EDTA buffer.

## Results

To address the role of Mg^2+^ in catalysis and specificity, we systematically studied the effects of free Mg^2+^ concentration on each step of the nucleotide incorporation pathway outlined in Figure 1: ground-state binding (*K*_*1*_), forward and reverse rates of the conformational change (*k*_*2*_ and *k*_*−2*_), chemistry (*k*_*3*_), and PPi release (*k*_*4*_). We began by measuring the rate and equilibrium constants governing nucleotide binding and enzyme conformational dynamics.

### Effect of free Mg^2+^ concentration on Mg.dTTP binding kinetics and equilibrium

**Figure 2:**
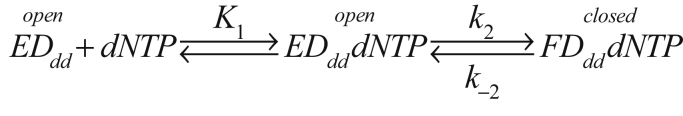
**Two-step nucleotide binding.** The simplified model shows only the binding and conformational change steps when chemistry is blocked by using a dideoxy-terminated DNA primer.

To study the kinetics and equilibria for binding of Mg.dTTP to HIV-RT, we used HIV-RT labeled with MDCC (7-diethylamino-3-((((2-maleimidyl)ethyl)amino)carbonyl) coumarin) on the fingers domain as described previously. The labeling provides a signal to measure the conformational changes between open and closed states (16). The fluorescence change was recorded using a stopped flow instrument after rapidly mixing various concentrations of Mg.dTTP with an enzyme–DNA complex formed with a dideoxy-terminated primer (ED_dd_) so that Mg.dTTP binds but does not react to mimic conditions used to solve crystal structures (21). The fluorescence signal is due to the fast closing of the enzyme after Mg.dTTP binding, but at low concentrations of nucleotide the rate is limited by the kinetics of binding, affording measurement of the net second-order rate constant for Mg.dNTP binding (Fig. 2).

Under conditions of rapid equilibrium substrate binding, the reaction follows a single exponential with an observed decay rate (eigenvalue, λ) that is a hyperbolic function of the substrate concentration. At low substrate concentrations, the slope of the concentration dependence defines the apparent second-order rate constant for substrate binding, *k*_on_.

(1) $$\begin{array}{l} Y=\;A_{0}\;+\;A_{1}\left(1-e^{-\lambda t}\right)\\ \lambda =\;\frac{K_{1}k_{2}\left[Mg.dNTP\right]}{K_{1}\left[Mg.dNTP\right]\;+\;1}\;+\;k_{-2}\\ k_{on}=\frac{d\lambda }{d\left[Mg.dNTP\right]}=K_{1}k_{2}\;\text{W}\text{h}\text{e}\text{n}\;\left[Mg.dNTP\right]<<1/K_{1}\\ k_{off}=k_{-2}\;K_{d}=k_{off}/k_{on} \end{array}$$

Because binding data were collected only at low nucleotide concentrations, the data did not resolve *K*_*1*_ and *k*_*2*_; rather, we only determined the apparent second-order rate constant for Mg.dNTP binding given by the product *K*_*1*_*k*_*2*_.

**Figure 3:**
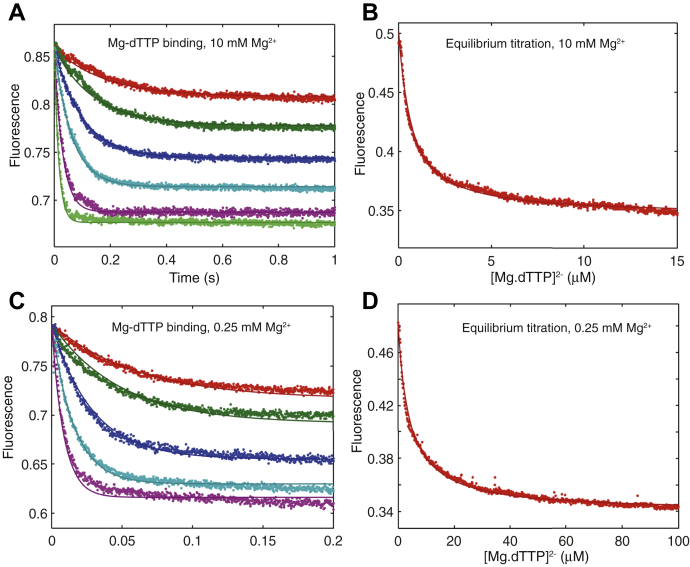
**Kinetics of Mg.dTTP binding.** The binding of Mg.dTTP was measured by stopped-flow fluorescence using a dideoxy-terminated primer to prevent chemistry (*A*). The experiments were performed by rapidly mixing various concentrations (0.5, 1, 2, 5, 10, and 20 μM) of Mg.dTTP with a preformed enzyme–_dd_DNA complex (100 nM MDCC-labeled HIV-1 wildtype RT and 150 nM DNA with a dideoxy-terminated primer) in the presence of 10 mM free Mg^2+^. The binding of Mg.dTTP to HIV-RT was also measured by equilibrium titration (*B*). The experiment was performed by titrating the preformed enzyme–DNA complex with varying concentrations of Mg.dTTP ranging from 0 to 20 μM. Global fitting of two experiments simultaneously to the model shown in Figure 2 allows us to accurately define the binding affinity and rate constants for association and dissociation of Mg.dTTP. The experiments in *A* and *B* were repeated in the presence of 0.25 mM Mg^2+^ to give the results shown in *C* and *D*. The results of data fitting are summarized in Table 1.

The kinetics of binding are shown at various concentrations of Mg.dTTP in Figure 3, *A* and *C* in the presence of 10 and 0.25 mM free Mg^2+^, respectively. It should be noted that the *K*_*d*_ for Mg^2+^ binding to nucleotide is 29 μM (9), so the Mg.dTTP complex is saturated even at the lowest concentrations of Mg^2+^ used in this study. The need to maintain saturation of the Mg.dTTP complex set a lower limit for the concentrations of free Mg^2+^ that we could explore. In Figure 3, *A* and *C*, the rate and amplitude of the reaction increase as a function of Mg.dTTP concentration so that both the forward and reverse rate constants can be defined from the data. That is, the rate of binding is a function of the sum of the forward and reverse rate constants, while the fluorescence endpoint defines the equilibrium constant, which gives the ratio of the rate constants. In global data fitting, the information is combined to define both forward and reverse rate constants (*k*_on_ = *K*_*1*_*k*_*2*_ and *k*_off_ = *k*_*−2*_). As a further test, we performed equilibrium titrations (Fig. 3, *B* and *D* for 10 and 0.25 mM Mg^2+^, respectively). Global fitting of the two experiments at each Mg^2+^ concentration using the model shown in Figure 2 allows us to accurately define the net dissociation constant for Mg.dTTP, *K*_*d*_ = *k*_off_/*k*_on_ = 1/(*K*_*1*_(1 + *K*_*2*_)) ≈ 1/*K*_*1*_*K*_*2*_, as well as the rate constants governing binding and dissociation as summarized in Table 1. From these results we concluded that the free Mg^2+^ concentration has little effect on the net nucleotide binding affinity. In the studies described below, we resolved these two constants (*K*_*1*_ and *k*_*2*_) using the full reaction with a normal DNA primer. The experiments reported here correlate our fluorescence signal with published structures and serve as a control for the effects of the dideoxy-terminated primer on the binding kinetics. In the next set of experiments, we measured the kinetics of reaction with a normal primer.

**Table 1:** Kinetic and equilibrium constants for Mg.dTTP binding to ED_d*d*_

[Mg^2+^] mM	*K*_*d*_ (μM)	*k*_on_ (μM^−1^ s^−1^)	*k*_off_ (s^−1^)
10	0.67 ± 0.01	6 ± 0.1	4 ± 0.06
0.25	1.2 ± 0.07	8.8 ± 0.5	9.7 ± 0.2

Rate and equilibrium constants were derived in globally fitting data in Figure 3, according to the scheme in Figure 2.

### Complete kinetic analysis at 0.25, 1, and 10 mM free Mg^2+^

**Figure 4:**
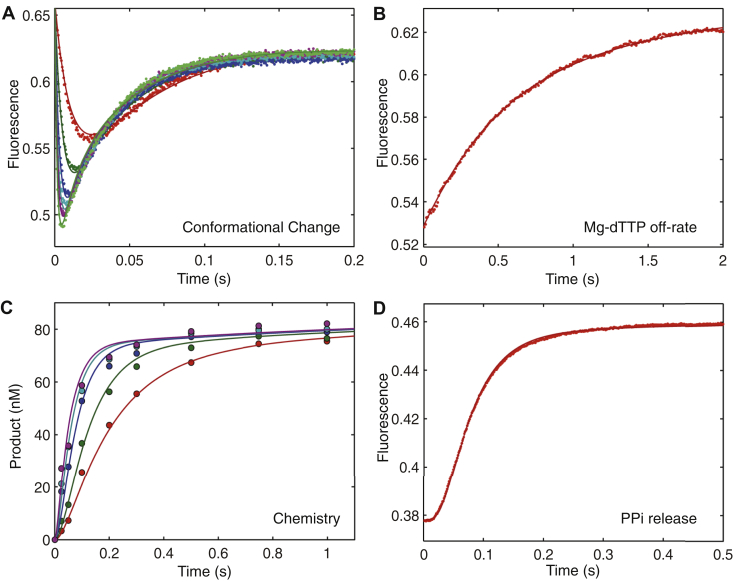
**Correct nucleotide binding and incorporation in the presence of 10 mM Mg**^**2+**^**.***A*, the time dependence of the fluorescence change upon dTTP incorporation was monitored by stopped-flow fluorescence. The experiment was performed by rapidly mixing preformed ED complex (100 nM) with various concentrations (10, 25, 50, 75, 100, and 150 μM) of dTTP. *B*, the nucleotide dissociation rate was measured by rapidly mixing preformed enzyme–DNA_dd_–dNTP complex (100 nM ED_dd_ complex, 1 μM nucleotide) with a nucleotide trap consisting of 2 μM unlabeled ED complex, and the fluorescence change was recorded to measure dNTP release. *C*, the rapid chemical quench-flow experiment was performed by rapidly mixing preformed ED complex (100 nM) with various concentrations (1, 2, 5, 10, and 20 μM) of dTTP. *D*, the rate of PPi release was measured by a coupled pyrophosphatase/phosphate sensor assay (20). Four experiments were fit simultaneously to define the kinetic parameters governing nucleotide incorporation as shown in Figure 1 and summarized in Table 2.

**Figure 5:**
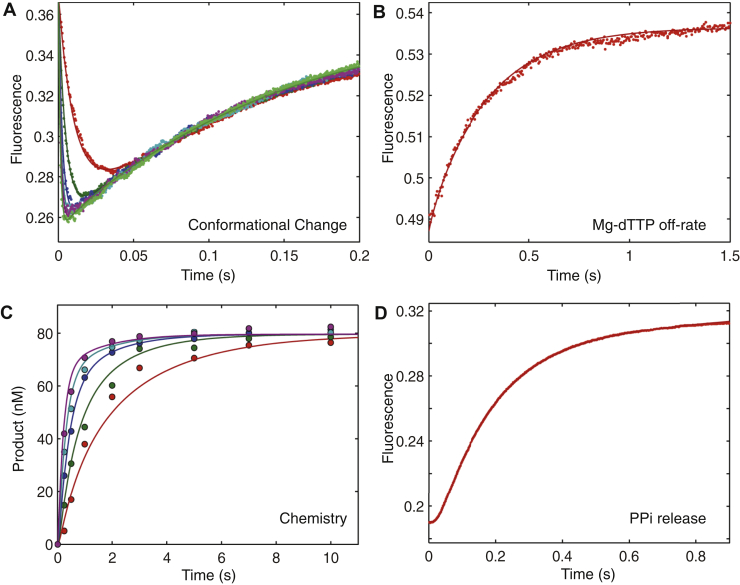
**Correct nucleotide binding and incorporation in the presence of 1 mM Mg**^**2+**^**.** Experiments were performed as described in Figure 4, but at 1 mM Mg^2+^. Four experiments were fit simultaneously to define each kinetic parameter governing nucleotide incorporation in the presence of 1 mM free Mg^2+^ as shown in Figure 1 and summarized in Table 2.

**Figure 6:**
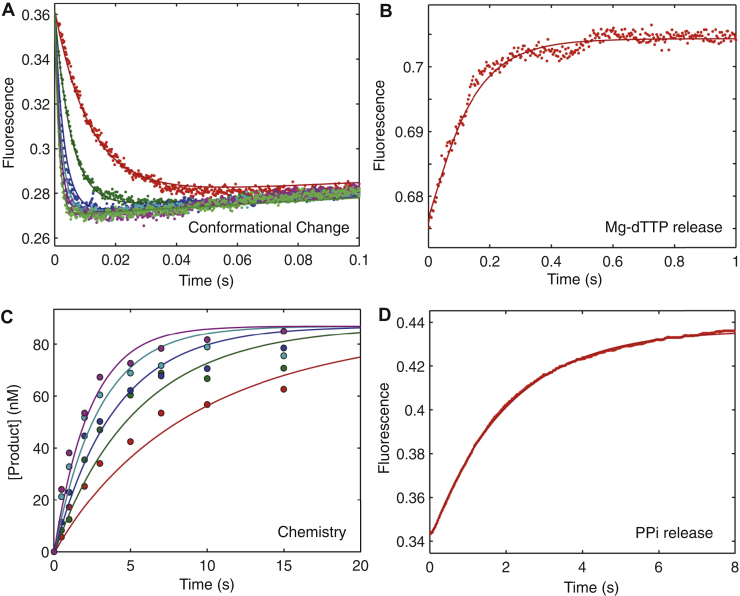
**Correct nucleotide binding and incorporation in the presence of 0.25 mM Mg**^**2+**^**.** Experiments were performed as described in Figure 4, but at 0.25 mM Mg^2+^, except that the method to measure nucleotide dissociation was modified owing to the low efficiency of the nucleotide trap at low Mg^2+^ concentration. We used a combination of two enzymes (unlabeled HIV-RT-DNA and apyrase) to trap and digest free nucleotides in solution (Fig. 6*B*). The *k*_*−2*_ value obtained by this method was 9.9 ± 0.4 s^−1^. Four experiments were fit simultaneously to define each kinetic parameter governing nucleotide incorporation in the presence of 0.25 mM free Mg^2+^ as shown in Figure 1 and summarized in Table 2.

To measure the effects of free Mg^2+^ concentration on each step of the nucleotide incorporation pathway, experiments to measure the kinetics of nucleotide binding and dissociation, enzyme conformational changes, chemistry, and PPi release were all fitted globally at each free Mg^2+^ concentration. Figure 4, Figure 5, Figure 6 show the results obtained at 10, 1, and 0.25 mM free Mg^2+^, respectively. Figure 4*A* shows the time dependence of the fluorescence change after mixing the enzyme–DNA complex with various concentrations of Mg.dTTP. In each single turnover experiment, the decrease in fluorescence is due to enzyme closing after nucleotide binding, whereas the increase in fluorescence is due to enzyme opening after chemistry.

To measure the rate of the reverse of the conformational change, the nucleotide dissociation rate was measured by rapidly mixing a preformed enzyme–DNA_dd_–dTTP complex (100 nM MDCC-labeled HIV-RT, 150 nM 25ddA/45 nt, 1 μM Mg.dTTP) with a nucleotide trap that consists of 2 μM unlabeled enzyme–DNA complex (Fig. 4*B*). Because the DNA primer was dideoxy terminated, premixing the ED_dd_ complex with the incoming nucleotide allows the binding but not the chemistry. After the addition of the large excess of the unlabeled enzyme–DNA complex, the rate of the fluorescence change defines the rate for the reverse of the conformational change, allowing rapid release of the nucleotide. Because these data were fit using computer simulation and we included the known kinetics of the nucleotide binding to the enzyme–DNA nucleotide trap, it was not necessary to repeat this experiment at multiple concentrations of the nucleotide trap. These results define Mg.dNTP binding and release and agree with the measurement of the net equilibrium constant shown in Figure 3, *B* and *D*.

In the next experiment, the rate of chemistry was measured in a rapid quench-flow assay (Fig. 4*C*). An enzyme–DNA complex (^32^P-labeled primer) was rapidly mixed with varying concentrations of incoming nucleotide. The rate of product formation as a function of nucleotide concentration was used to define the maximum polymerization rate (*k*_cat_) of the reaction and the specificity constant (*k*_cat_/*K*_*m*_). The data in Figure 4 (as well as Figs. 5 and 6) were fit globally using Figure 1. The estimated rate constants were used to calculate *k*_cat_ and *k*_cat_*/K*_*m*_ using Equation 4 (Experimental procedures).

To measure the rate of PPi release, a coupled pyrophosphatase/phosphate sensor assay was performed as described (22, 23). Because there is a large fluorescent change upon phosphate binding to MDCC-PBP (MDCC-labeled phosphate binding protein) and the rate of phosphate binding to MDCC-PBP is much faster than that of the PPi release from HIV-RT (24), the time course of the fluorescent change defines the rate of PPi release (22). Our data show that the rate of PPi release was coincident with the rates of chemistry and of reopening the enzyme as measured by the signal from fluorescently labeled HIV-RT. In modeling the data by computer simulation, a lower limit on the rate of the PPi release was set at >5-fold faster than the rate of the chemistry (Table 2), assuming opening was followed by PPi release. In fitting the data by simulation, the minimum rate of PPi release was defined as the value sufficient to make the two processes appear to coincide. Of course, we do not know the order of PPi release and enzyme opening because the two signals are coincident.

**Table 2:** Kinetic constants for Mg.dTTP binding, conformational change, incorporation, and PPi releas*e*

[Mg^2+^] (mM)	1/*K*_1_ (μM)	*k*_2_ (s^−1^)	*k*_−2_ (s^−1^)	*K*_2_	*k*_3_ (s^−1^)	*k*_4_ (s^−1^)
10	275 ± 3	2000 ± 67	3.9 ± 0.1	513 ± 22	21 ± 0.1	>100
1	213 ± 3	1960 ± 173	9.4 ± 0.2	209 ± 19	6 ± 0.1	>160
0.25	216 ± 2	1910 ± 178	9.7 ± 0.2	197 ± 19	0.6 ± 0.01	>20

**Figure 7:**
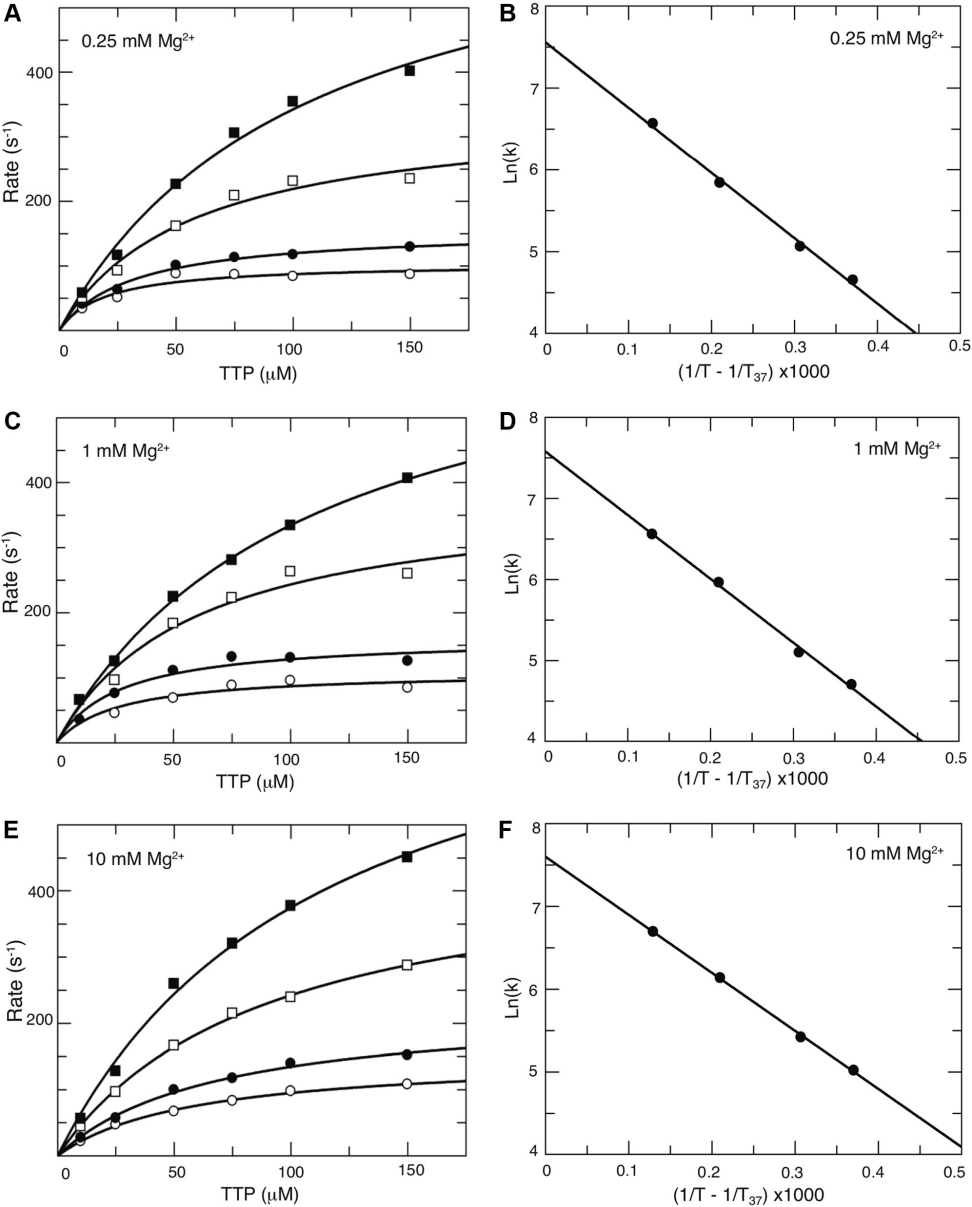
**Mg**^**2+**^**does not affect the rate of the conformational change.** In order to estimate the rate of the conformational change at 37 °C, the temperature dependence of the fluorescence change after dTTP binding was measured by stopped-flow methods at 0.25 (*A*–*B*), 1 (*C*–*D*), and 10 mM (*E*–*F*) free Mg^2+^ concentration in buffer containing 50 mM Tris pH 7.5 and 100 mM potassium acetate. At each Mg^2+^ concentration, an enzyme–DNA complex (50 nM) was rapidly mixed with various concentrations of dTTP (10, 25, 50, 75, 100, and 150 μM) at 5 °C (○), 10 °C (●), 18 °C (□), and 25 °C (▪) (*A*, *C*, and *E*). The concentration dependence of the rate of the fluorescence decrease upon nucleotide binding was fit to a hyperbolic equation to obtain the maximum rate of the conformational change (*k*_*2*_) at each temperature. The temperature dependence of *k*_*2*_ was then analyzed on an Arrhenius plot (*B*, *D*, and *F*) to estimate the maximum rate of the conformational change at 37 °C at each free Mg^2+^ concentration. The data show that the rate of the conformational change is independent of Mg^2+^ concentration (Tables 2 and 3).

Rate and equilibrium constants were derived in globally fitting data in Figure 4, Figure 5, Figure 6, Figure 7 as described in the Results. Rate constants refer to Figure 1.

The four experiments were fit simultaneously to rigorously define kinetic parameters governing nucleotide incorporation (Fig. 1) to get the results summarized in Table 2. To investigate the effects of free Mg^2+^ concentration on each step of the pathway, similar experiments and analyses were repeated at 1 and 0.25 mM free Mg^2+^ as shown in Figures 5 and 6, respectively.

The kinetic parameters are summarized in Table 2. The results show that Mg^2+^ concentrations (from 0.25 to 10 mM) do not significantly affect the ground-state Mg.dNTP binding (*K*_*1*_) or the rate of the conformational change (*k*_*2*_) but greatly affect the rate of the chemistry (*k*_*3*_) and less so the reverse of the conformational change (or enzyme reopening) (*k*_*−2*_). Although it appears that Mg^2+^ concentrations affect the rate of PPi release, this reflects the rates of chemistry—our simulation only gives a lower limit and therefore no direct measurement of the PPi release rate was possible. Our data support the conclusion that PPi release was not rate limiting at any of the Mg^2+^ concentrations examined.

### Free Mg^2+^ concentrations do not affect the rate of the conformational change step

The experiments shown in Figure 4, Figure 5, Figure 6 are not sufficient to resolve the rate of the conformational change step because it is too fast to measure directly at 37 °C. Therefore, we measured the concentration dependence of the fluorescence transient at several temperatures and then extrapolated the observed rate constant (*k*_*2*_) to estimate the value at 37 °C. The enzyme–DNA complex was rapidly mixed with various concentrations of dTTP as described in Figures 4*A* and 5A, and 6A but repeated at temperatures of 5, 10, 18, and 25 °C. At each dTTP concentration the fluorescence transient was biphasic and was fit to a double exponential function:

(2) $$\begin{array}{l} Y=A_{1}e^{-\lambda_{1}t}\;+\;A_{2}e^{-\lambda_{2}t}\;+\;c\\ \lambda_{1}=\frac{K_{1}k_{2}\left[MgdNTP\right]}{1\;+\;K_{1}\left[MgdNTP\right]}\;+\;k_{-2}\;+\;k_{3} \end{array}$$

The concentration dependence of the rate of the fast phase of the fluorescence transient was then fit to a hyperbolic equation to obtain the maximum rate of the observed conformational change (λ_max_ = *k*_*2*_ *+ k*_*−2*_ *+ k*_*3*_) at each temperature. Note that *k*_*−2*_ and *k*_*3*_ are much smaller than *k*_*2*_ so λ_max_ ≅ *k*_*2*_. The experiments were repeated at various concentrations of free Mg^2+^: 0.25 mM (Fig. 7*A*), 1 mM (Fig. 7*C*), and 10 mM (Fig. 7*E*). The observed maximum decay rate (λ_max_) observed at each temperature was then graphed on an Arrhenius plot (Fig. 7, *B*, *D* and *F*) and the values of *k*_*2*_ at 37 °C at each of three different free Mg^2+^ concentrations were obtained by extrapolation by linear regression. The results indicate that Mg^2+^ concentrations (from 0.25 to 10 mM) do not affect the rate constant for the conformational change (*k*_*2*_) (Tables 2 and 3). These results imply that Mg^2+^ binding from solution (at a concentration greater than 0.25 mM) is not required for the conformational change step, but it is required for the chemical reaction. By combining the rate constant for the conformational change (*k*_*2*_) with the estimates of *k*_*−2*_ from the nucleotide dissociation rate, we can calculate the equilibrium constant for the conformational change step (*K*_*2*_, Table 2).

**Table 3:** Temperature dependence of HIV-RT conformational change at various concentrations of free Mg^2*+*^

[Mg^2+^] (mM)	*k*_*2*_ value at 5 °C, (s^−1^)	*k*_*2*_ value at 10 °C, (s^−1^)	*k*_*2*_ value at 18 °C, (s^−1^)	*k*_*2*_ value at 25 °C, (s^−1^)
0.25	104 ± 10	157 ± 7	343 ± 34	707 ± 69
1	110 ± 12	163 ± 14	388 ± 56	702 ± 27
10	150 ± 6	225 ± 13	461 ± 17	805 ± 68

Kinetic parameters were derived in fitting data in Figure 7 as described in the Results.

### The net K_d_ for Mg.dTTP binding

The substrate, Mg.dTTP, binds initially to the open state with a relatively weak affinity, *K*_*d*_ = 275 μM at 10 mM Mg^2+^, which is followed by the conformational change leading to a much tighter nucleotide binding. The net *K*_*d*_ for the two-step binding is defined by:

$$K_{d,net}=\frac{1}{K_{1}\left(1+K_{2}\right)}$$

Accordingly, the net *K*_*d*_ for Mg.dTTP binding at 10 mM Mg^2+^ was 0.5 μM and increased to values of 1.0 and 1.1 μM at 1 and 0.25 mM Mg^2+^, respectively (Table 2). Thus, the nucleotide binding gets slightly tighter as the Mg^2+^ concentration increases, but this net effect is a product of opposing effects in the two-step binding, which we explore further below.

### Mg^2+^ concentration dependence of chemistry

**Figure 8:**
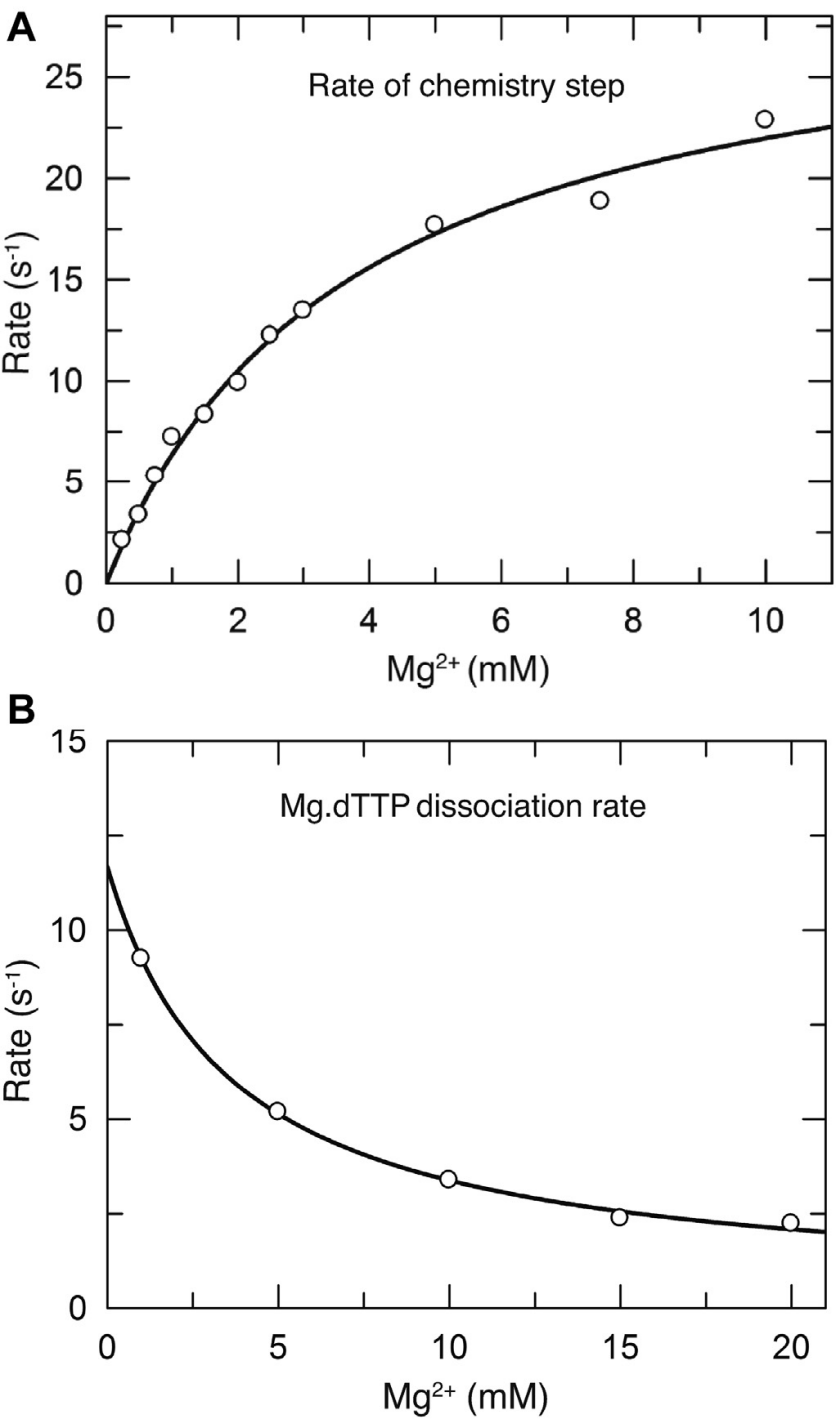
**Mg**^**2+**^**concentration dependence of the rates of chemistry and nucleotide release.***A*, Mg^2+^ dependence of the rate of chemistry was measured by mixing an ED complex with a fixed concentration of Mg.dTTP (150 μM) at various concentrations of free Mg^2+^, ranging from 0.25 to 10 mM. The reaction was then quenched with 0.5 M EDTA at various times, and the amount of product formed was quantified and fit to a single exponential function. The measured rates were then plotted as a function of free Mg^2+^ concentration and fit to a hyperbola to obtain the maximal rate of chemistry and the apparent dissociation constant (*K*_*d, app*_ = 3.7 ± 0.1 mM) for Mg^2+^ in stimulating the enzyme to catalyze the reaction. *B*, Mg^2+^-dependent rates of nucleotide release were measured by rapidly mixing a preformed E-DNA_dd_-dTTP complex (100 nM ED_dd_ complex, 1 μM dTTP) with a nucleotide trap consisting of 2 μM unlabeled ED complex at various Mg^2+^ concentrations, ranging from 1 to 20 mM. The release of dTTP from the ED_dd_ complex was monitored by stopped-flow fluorescence to define the rate of nucleotide release. The measured rates were then plotted as a function of free Mg^2+^ concentration and fit to a hyperbola to obtain and apparent *K*_*d*_ = 3.7 ± 0.2 mM for Mg^2+^ in slowing the rate of nucleotide release.

The apparent binding affinity for the catalytic Mg^2+^ was measured more accurately by examining the Mg^2+^ concentration dependence of the rate of catalysis in a single turnover experiment. The experiment was performed by mixing an ED complex with solutions containing a fixed concentration of Mg.dTTP (150 μM) and various concentrations of free Mg^2+^ (ranging from 0.25 to 10 mM). This concentration of Mg.dTTP is much greater than its *K*_*m*_, so we are measuring the rate of the chemistry step in this experiment. The rates of chemistry, measured by rapid-quench and stopped-flow fluorescence methods, were observed for each reaction and plotted as a function of free Mg^2+^ concentrations (Fig. 8*A*). The data were fit to a hyperbola to derive an apparent dissociation constant, *K*_*d,app*_ = 3.7 ± 0.1 mM, for the catalytic Mg^2+^. Because other known Mg^2+^ binding events reach saturation at much lower concentrations of Mg^2+^, they do not affect the measured *K*_*d*_ for the catalytic Mg^2+^. For example, the dissociation constant for the formation of the Mg.dTTP complex (28.7 μM) was 130-fold lower than the net *K*_*d*_ for binding of the catalytic Mg^2+^ (3.7 mM). Therefore, the observed concentration dependence of catalysis reflects only the binding of the catalytic Mg^2+^ to the closed ED–Mg.dTTP complex.

### Binding of the catalytic Mg^2+^ also affects the reverse of the conformational change

In Figure 8*B* we show the Mg^2+^ concentration dependence of the nucleotide dissociation rate, which we believe is limited by the rate of enzyme opening (*k*_*−2*_). We plotted the observed rate of nucleotide dissociation as a function of free Mg^2+^ concentration and fit the data to a hyperbola to provide an estimated *K*_*d*_ = 3.7 ± 0.2 mM. This defines the apparent *K*_*d*_ for the binding of Mg^2+^ to decrease the rate of nucleotide dissociation. The Mg^2+^ dependence of the nucleotide dissociation rate parallels the concentration dependence of the observed rate of the chemical reaction (Fig. 8*A*) suggesting that the two effects are due to the same Mg^2+^ binding event. The binding of the catalytic Mg^2+^ stabilizes the closed enzyme state as active site residues are aligned to carry out catalysis. Although the effect of Mg^2+^ binding on the rate of chemistry is profound (from <0.1 to 25 s^−1^), there is only a modest decrease (∼2-fold) in the rate of nucleotide dissociation as the Mg^2+^ concentration is increased from 0.25 to 10 mM.

### Catalytic Mg^2+^ is not required for the enzyme closing

**Figure 9:**
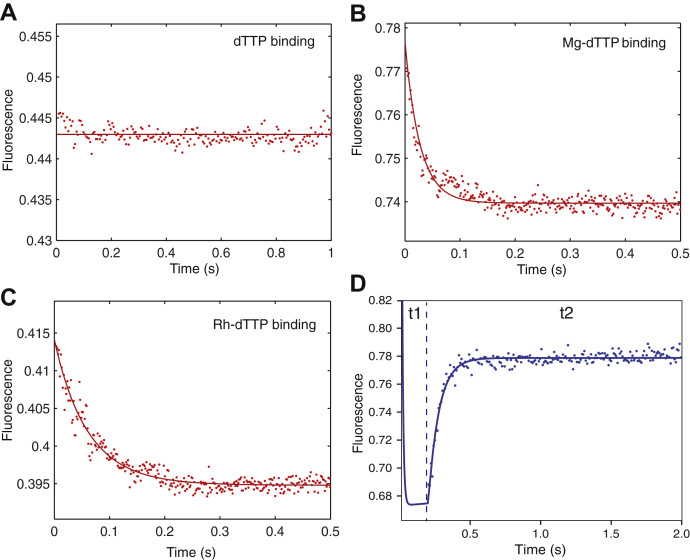
**Role of Mg**^**2+**^**in nucleotide binding–induced conformation change and catalysis.** The role of Mg in the enzyme nucleotide-induced conformational change was examined using MDCC-HIV-RT fluorescence under various conditions with a preformed ED_dd_ complex (100 nM). *A*, we rapidly mixed ED_dd_ with dTTP (50 μM) in the absence of Mg^2+^ (concentration of EDTA: 500 μM). *B*, we rapidly mixed ED_dd_ with Mg.dTTP (50 μM) (concentrations of Mg.dTTP was controlled by using EDTA and simulated by using *KinTek Explorer* software). *C*, we rapidly mixed ED_dd_ with Rh-dTTP (50 μM) in the absence of Mg^2+^. *D*, a double mixing experiment was performed by first mixing an ED complex (100 nM) with 10 μM Mg.dTTP in the presence of 25 μM free Mg^2+^ for 0.2 s (t1), followed by second mixing with 10 mM Mg^2+^ (t2). The fluorescence increase after the secondary mixing was monitored by the stopped-flow assay showing the fast opening of the enzyme after chemistry.

Our results imply that the Mg.dTTP alone is sufficient for nucleotide binding and enzyme closing because the rate of conformational change (*k*_*2*_) is independent of free Mg^2+^ concentration. To further test this postulate, a preformed ED_dd_ complex (100 nM MDCC-labeled HIV-RT and 150 nM 25ddA/45 nt DNA) was rapidly mixed with either 50 μM dTTP or Mg.dTTP. The change of fluorescence upon dTTP or Mg.dTTP binding was monitored by stopped flow methods. In each experiment, we added 50 μM dTTP, but the free Mg^2+^ concentration was controlled using EDTA to allow the formation of 50 μM Mg.dTTP or ∼0 μM Mg^2+^ to give free dTTP. The results showed that Mg.dTTP but not dTTP induces the conformational change of HIV-RT (Fig. 9, *A*–*B*). However, one could still argue that the trace of Mg^2+^ needed to form Mg.dTTP could influence the observed conformational change kinetics. As a further test, we examined the kinetics of the fluorescence change after adding Rh-dTTP, an exchange-inert metal-nucleotide complex. In the absence of free Mg^2+^, 50 μM Rh-dTTP induced a decrease of the fluorescence (Fig. 9*C*), although slightly lower in rate and amplitude when compared with Mg.dTTP. These results indicate that the metal–nucleotide complex is sufficient to induce enzyme closing. However, the lower rate and amplitude seen with Rh-dTTP compared with Mg.dTTP reveals differences between the two metal ion complexes. These results support conclusions derived from analysis in Figure 4, Figure 5, Figure 6, Figure 7 (summarized in Tables 2 and 3) suggesting that the concentration of free Mg^2+^ does not alter the rate of the conformational change step.

Because the catalytic Mg^2+^ binds relatively weakly, we can easily resolve effects of the metal ions on the conformational change *versus* chemistry by varying the Mg^2+^ concentration in the experiment. We also examined whether the catalytic Mg^2+^ participated in the conformational change by using a double mixing experiment (Fig. 9*D*). The experiment was performed by first mixing the ED complex (100 nM MDCC-labeled HIV-RT and 150 nM 25/45 nt DNA) with 10 μM Mg.dTTP in the presence of 25 μM free Mg^2+^ for 0.2 s (t1), followed by a second mixing with a large excess of free 10 mM Mg^2+^ (t2). The fluorescence change upon the second mixing was monitored by stopped-flow fluorescence to measure the opening of the enzyme after chemistry. During the first mixing step at 25 μM free Mg^2+^ the half-life of dTTP incorporation was >4 s (Fig. 4*D*); therefore, chemistry did not occur significantly during the first mixing step of 0.2 s. The apparent *K*_*d*_ = 3.7 mM predicts that only 0.7% of the catalytic Mg^2+^-binding sites will be occupied at 25 μM Mg^2+^. During the second mixing with the addition of a large excess of free Mg^2+^ (10 mM), the catalytic Mg^2+^ binds to HIV-RT and stimulates catalysis, which is followed by rapid opening of the enzyme to give a fluorescence signal. These data demonstrate that Mg.dTTP is sufficient to induce enzyme closing without the catalytic Mg^2+^. If the catalytic Mg^2+^ were required for the closing of the enzyme, we would have observed a decrease in fluorescence followed by an increase after adding excess Mg^2+^ in the second mixing step. The immediate reopening of the enzyme directly after the second mixing (Fig. 9*D*) demonstrates that Mg.dTTP alone is sufficient to induce the conformational change from the *open* to the *closed* state of HIV-RT. The binding of the catalytic Mg^2+^ in the second mixing step is necessary for fast catalysis and enzyme opening but is not required for the conformational change step. The catalytic Mg^2+^ binds only after enzyme closing to stimulate catalysis.

### Mg^2+^ binding to the open state of the enzyme

With a pair of aspartic acid residues in the active site, one might expect that Mg^2+^ could bind tightly to the open state of the enzyme in the absence of nucleotide, but this is never seen in crystal structures. We reasoned that, if Mg^2+^ binds to the open state of the enzyme–DNA complex, then it could be a competitive inhibitor of Mg.dNTP binding. A slight effect can be seen in the data in Table 2. Values of the apparent *K*_*d*_ for Mg.dNTP in the ground-state binding to the open form of the enzyme (1/*K*_*1*_) increase as the concentration of Mg^2+^ increases. Although we have only three data points because of the extensive analysis required to derive this number, the data can still provide an estimate of the *K*_*d*_ for Mg^2+^ binding based on the observed competition according to the following relationship:

$$K_{N,app}=K_{N}\left(1+\left[Mg\right]/K_{Mg}\right)$$

where *K*_*N*_ and *K*_*Mg*_ are the *K*_*d*_ values for Mg.dTTP and Mg^2+^, respectively, and *K*_*N,app*_ is the apparent *K*_*d*_ for nucleotide binding (given in Table 2). Linear regression of a plot of *K*_*N,app*_ *versus* [Mg] gives an estimate of *K*_*N*_ = 212 ± 4 μM and *K*_*Mg*_ = 34 ± 4 mM. This analysis suggests that the binding of Mg^2+^ to the open state of the enzyme in the absence of Mg.dTTP is 10-fold weaker than the binding to the closed state in the presence of Mg.dNTP.

Alternatively, our data are also consistent with a model invoking the formation of a Mg_2_dTTP, which does not bind to the enzyme, but its formation reduces the concentration of Mg.dTTP thereby reducing the observed apparent affinity. This postulate is based on kinetic analysis of hexokinase steady-state turnover as a function of Mg^2+^ and ATP concentrations, which provided evidence for the formation of a Mg_2_ATP complex with a *K*_*d*_ ≈ 25 mM, with no evidence of enzyme inhibition by the direct binding of Mg^2+^ to the enzyme (25). Regardless of the mode of observed inhibition, available structural and kinetic data support the postulate that the binding of Mg^2+^ to the open form of the enzyme is weak, at least to the extent to which it has no effect on the observed rate of the conformational change step. Below, we explore this hypothesis further using MD simulation methods.

### Mg^2+^ concentration effects on nucleotide specificity

The specificity constant (*k*_cat_/*K*_*m*_) defines the fidelity for nucleotide incorporation in comparing a cognate base pair with a mismatch. The specificity constant is best understood as the second-order rate constant for substrate binding times the probability that, once bound, the substrate goes forward to form and release product. Steady-state kinetic parameters were calculated from the primary rate constants (Equation 4, Experimental Procedures) to get the results summarized in Table 4. Our results showed that the value of the specificity constant is decreased by 12-fold as the free Mg^2+^ concentration is reduced from 10 to 0.25 mM owing to the slower rate of incorporation and change in the identity of the specificity-determining step; that is, nucleotide specificity is redefined as the free Mg^2+^ concentration is altered. Because the rate of the conformational change (*k*_*2*_) is much faster than chemistry (*k*_*3*_), the specificity constant depends on the kinetic partitioning governed by the relative values of *k*_*−2*_ *versus k*_*3*_ (16, 19). If *k*_*−2*_ >> *k*_*3*_, the ground-state binding and conformational change come to equilibrium and the specificity constant is governed by the product of binding equilibria and the rate of chemistry (*k*_cat_/*K*_*m*_ = *K*_*1*_*K*_*2*_*k*_*3*_). If *k*_*−2*_ << *k*_*3*_, the nucleotide binding fails to reach equilibrium during turnover and the rate of chemistry does not contribute to the specificity constant; rather, it is defined only the binding and conformational change steps (*k*_cat_/*K*_*m*_ = *K*_*1*_*k*_*2*_). As the Mg^2+^ concentration decreases, the rate of dissociation increases slightly, while the rate of chemistry decreases significantly. With the correct nucleotide (dTTP) incorporation at 0.25 mM Mg^2+^, the value of *k*_*−2*_ (9.7 ± 0.2 s^−1^) is much greater than that of *k*_*3*_ (0.6 ± 0.01 s^−1^), suggesting that the *k*_cat_/*K*_*m*_ value is governed by the product of binding equilibrium constants and the rate of chemistry (*k*_cat_/*K*_*m*_ = *K*_*1*_*K*_*2*_*k*_*3*_). When the free Mg^2+^ concentration was increased to 10 mM, the value of *k*_*−2*_ (3.9 ± 0.1 s^−1^) is less than that of *k*_*3*_ (21 ± 0.1 s^−1^) and therefore *k*_cat_/*K*_*m*_ is largely governed only by the nucleotide binding (*k*_cat_/*K*_*m*_ = *K*_*1*_*k*_*2*_). Thus, the mechanistic basis for nucleotide specificity changes as a function of the free Mg^2+^ concentration. We have not performed detailed analysis of the kinetics of incorporation at the lower Mg^2+^ concentrations because when the Mg^2+^ concentration is less than 0.25 mM the dNTP is not saturated with Mg^2+^ leading to more complex effects, including inhibition by free dNTP.

**Table 4:** Steady-state kinetic parameters *versus* concentration of free magnesium io*n*

[Mg^2+^] (mM)	*K*_*d,net*_ (μM)	*K*_*m*_ (μM)	*k*_cat_ (s^−1^)	*k*_cat_*/K*_*m*_ (μM^−1^ s^−1^)	Fold change in *k*_cat_*/K*_*m*_
10	0.5 ± 0.02	3.6 ± 0.1	20.7 ± 1	6 ± 0.3	1
1	1.0 ± 0.09	1.7 ± 0.2	6 ± 0.7	3.5 ± 0.6	0.6
0.25	1.1 ± 0.1	1.2 ± 0.1	0.6 ± 0.08	0.5 ± 0.08	0.08

The steady-state and equilibrium constants were calculated as described in the Methods.

**Figure 10:**
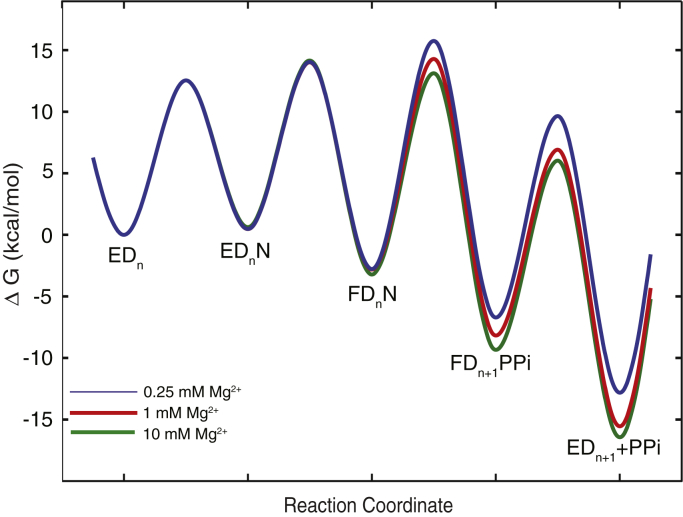
**Free-energy profile for nucleotide binding and catalysis at various Mg**^**2+**^**concentrations.** The free-energy diagrams for dTTP incorporation at 0.25, 1, and 10 mM free Mg^2+^ concentrations are shown in *blue*, *brown* and *green*. The free energy was calculated as ΔG = RT[ln(*k*T/h)-ln(*k*_*obs*_)] kcal/mol using rate constants derived from global fitting, where the constant k is the Boltzmann constant, T is 310 K, h is Planck’s constant, and *k*_*obs*_ is the first-order rate constant for each step. The nucleotide concentration was set equal to 100 μM to calculate *k*_*obs*_ as the pseudo-first-order rate constant for nucleotide binding. Nucleotide binding to the open state (ED) was assumed to be diffusion limited with *k*_*1*_ = 100 μM^−1^ s^−1^.

We can illustrate the effects of free Mg^2+^ on free energy profiles of governing nucleotide incorporation at three different free Mg^2+^ concentrations (0.25, 1, and 10 mM) as shown in Figure 10. The *k*_cat_/*K*_*m*_ value is determined by the energy barrier between its highest peak relative to its unbound state. At 0.25 mM free Mg^2+^, the highest peak is the state between FD_n_N and FD_n+1_PPi (or chemistry step). Therefore, the nucleotide specificity is determined by all of the steps from its unbound state to the chemistry (*k*_cat_/K_m_ = *K*_*1*_*K*_*2*_*k*_*3*_). At 10 mM free Mg^2+^, the highest peak is the state between ED_n_N and FD_n_N (or conformational change step). Thus, nucleotide specificity is determined by only two steps including ground-state binding and the conformational change (*k*_cat_/*K*_*m*_ = *K*_*1*_*k*_*2*_). At 1 mM free Mg^2+^, the highest peak is not obvious by inspection, and therefore, a simplified equation for defining nucleotide specificity cannot be applied. To accurately define the nucleotide specificity constant (*k*_cat_/*K*_*m*_), the complete equation (Equation 4) containing each parameter has to be used. The free energy profiles showed that nucleotide specificity is redefined as the free Mg^2+^ concentration is altered from 10 to 0.25 mM.

Because the *K*_*d*_ of catalytic Mg^2+^ is higher than the intracellular concentration, our results suggest that binding of the catalytic Mg^2+^ provides the final checkpoint for nucleotide specificity as one component of the kinetic partitioning of the closed ED-Mg.dNTP complex so the substrate either dissociates or reacts to form products.

### Relating kinetics to available structures

**Figure 11:**
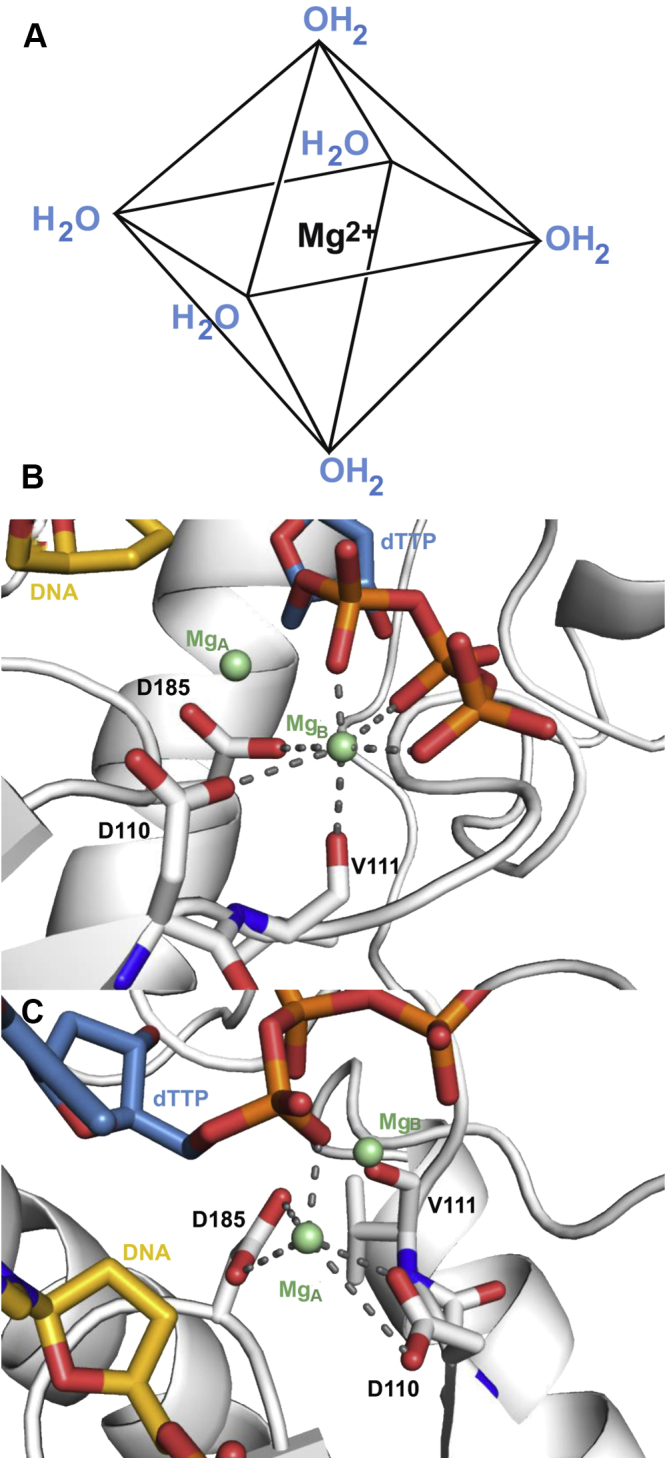
**HIV-RT magnesium coordination geometry.***A*, the octahedral coordination geometry is ideal for magnesium ions. Magnesium preferentially forms six evenly spaced polar interactions, demonstrated here as interactions with solvent waters. *B*, Mg_B_ (*pale green*) at the HIV-RT polymerase domain exhibits ideal coordination geometry by forming three polar contacts with active site residues aspartate 110, 185 and valine 111 (*white*), and three polar contacts with the triphosphate group of the substrate dTTP (*steel blue*) (Protien Data Bank ID 1RTD). *C*, Mg_A_ (*pale green*) at the HIV-RT polymerase domain exhibits atypical coordination geometry and forms four polar contacts with the side chains of aspartate 110 and 185 (*white*) and one polar contact to the triphosphate group of substrates dTTP (*steel blue*) (Protein Data Bank ID 1RTD).

In light of our kinetic results, we analyze the published structures with the focus on the coordination and interaction around Mg^2+^ ions. Magnesium prefers an octahedral coordination and will be most tightly bound when this geometry is satisfied (Fig. 11*A*). Mg^2+^ ions at the polymerase domain of HIV-RT are coordinated through polar interactions with the side chains of aspartates 110 and 185 and the three phosphates of the nucleotide substrate (Fig. 11, *B*–*C*). In the nucleotide-bound Mg^2+^ (Mg_B_ in Fig. 11, *B*–*C*), the carboxylate side chains of Asp110 and 185 as well as the two nonbridging oxygens from the phosphates of the nucleotide appear to form the four coordination interactions on the plane with a distance around 2.2 to 2.4 Å. Another phosphate oxygen and the carbonyl oxygen of valine 111 are at the apex from the opposite sides with a distance close to 2.6 Å. Therefore, Mg_B_ displays a classic octahedral coordination geometry (Fig. 11*B*). On the other hand, the catalytic Mg^2+^ (Mg_A_ in Fig. 11, *B*–*C*) deviates from the standard octahedral coordination with what appears as four coordination by the side chains of Asp110 and Asp185 but not forming a plane. The two apex coordination sites are occupied by nucleotide on one end but empty on the other (Fig. 11*C*), which is presumably occupied by a solvent water molecule that is not seen in the structure. The analysis of the metal coordination indicates that the two magnesium ions are bound differently, at least as observed by the refined crystal structures.

To understand the relative occupancy and mobility of the ions, we scrutinized the temperature factors for each ion. The temperature factor (or thermal factor, B-factor) is defined as a measure of deviation of an atom from the average position. A high B-factor correlates to high movement and low occupancy. Within the same molecule, the B-factor can be used to estimate the relative mobility of the atoms. The B-factors when both Mg^2+^ ions are present indicate that the mobility of the ions varies in HIV-RT. For the Protein Data Bank ID 4PQU in which crystals were formed at high Mg^2+^ concentration (10 mM), Mg_A_ and Mg_B_ exhibit comparable B-factors (30.69 *versus* 35.75 Å^2^, respectively). In other crystallization conditions in which HIV-RT was examined at lower concentrations of Mg^2+^ or with low-resolution diffraction, only one Mg^2+^ can be modeled in the density, which is consistently Mg_B_ (Table 5). In particular, Mg_A_ is poorly coordinated and displays a high relative B-factor or is missing in the structure. These structural data are in line with the measurement of the weak binding of *K*_*d*_ ≈ 3.7 mM for the catalytic Mg^2+^ observed in our solution study. In addition, no strong Mg^2+^ density was observed in the structure Protein Data Bank ID 3KJV in spite of the high Mg^2+^ ion concentration of 10 mM. In this structure, HIV-RT is complexed with DNA:DNA primer/template only, consistent with our estimate of very weak binding of Mg^2+^ to the open ED complex without the Mg.dNTP. This analysis provides a structural evidence to support our kinetic analysis concluding that Mg.dNTP binds first to induce enzyme closing and the catalytic Mg^2+^ binds weakly and is only seen in the closed state.

**Table 5:** Analysis of HIV-RT crystal structures

Structure components	Bound ligand	PDB ID	Mg_B_ B-Factor (Å^2^)	Mg_A_ B-Factor (Å^2^)	[Mg^2+^] in crystal drop (mM)
HIV-RT:DNA:DNA	NA	3KJV	NA	NA	2.5
HIV-RT:RNA:DNA	Mg-dATP	4PQU	30.7	35.8	10
HIV-RT:DNA:DNA	Mg-dATP	3KK2	5.1	NA	2.5
HIV-RT:DNA:DNA	Mg-AZTTP	3V4I	87.3	NA	10
HIV-RT:DNA:DNA	Mg-TFV	1T05	16.2	NA	10
HIV-RT:DNA:DNA	GS-9148 -phosphate	3KK1	15.9	NA	2.5

Published structures (cited in the table) were analyzed to quantify the binding of the nucleotide-bound Mg and catalytic Mg (MgA and MgB, respectively, as labeled in Figure 11).

### MD simulations to refine our understanding

Despite the significant evidence from kinetic analysis and structural data listed above suggesting a weaker binding and more rapid exchange of the catalytic Mg (Mg_A_), our solution study lacks atomic details. Structural studies provide valuable information at the atomic level but do not provide kinetic and thermodynamic parameters that define the role of Mg^2+^. In addition, it is naive to think that Mg^2+^ only binds to the tight-binding, static sites seen in the crystal structures as there are many electronegative sites available to attract positively charged ions. To fill this void and complement our kinetic studies, we performed MD simulations to gain further insights into metal ion coordination along the pathway of the DNA polymerase. Both matched and mismatched nucleotides can be studied to understand the role of metal ions in the enzyme’s function. Computer simulations of Mg^2+^ coordination provide molecular-level details that we cannot observe directly. However, we checked the validity of the MD simulations by comparison with what we can measure, Mg^2+^ binding affinities in the open and closed states, before and after Mg.dNTP binding, respectively.

**Figure 12:**
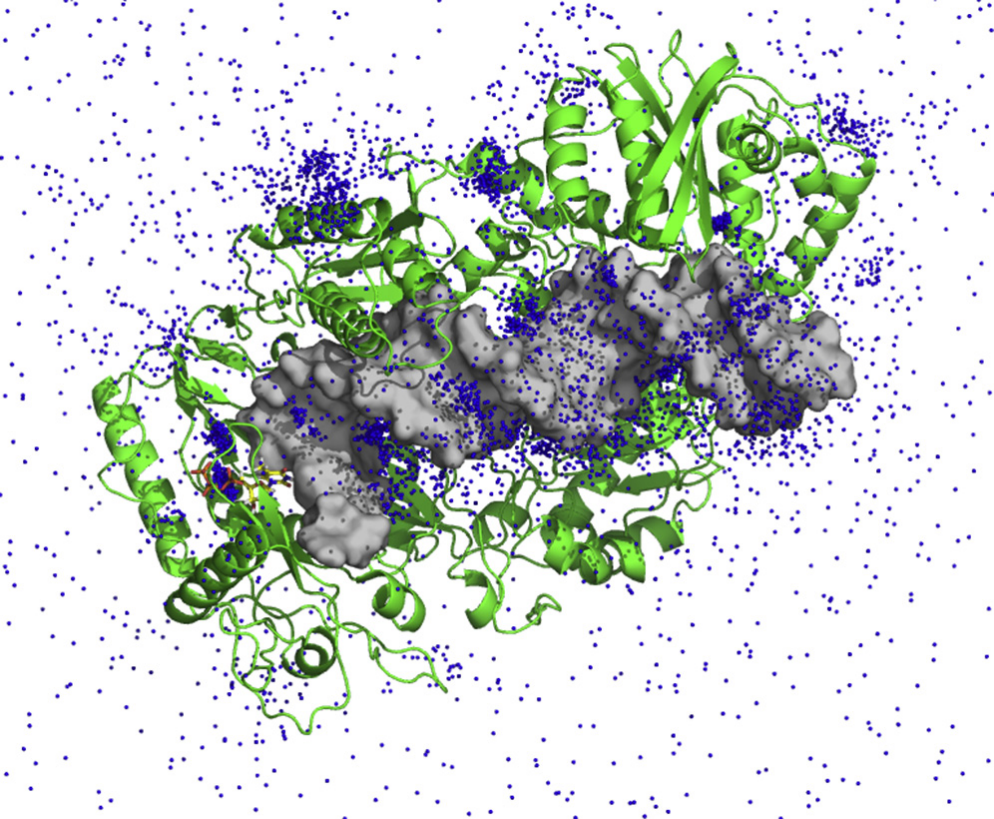
**Mg ion distribution from simulations.** Mg^2+^ ion positions sampled every 1 ns are combined and represented as dots to give a visual image of the probability density distributions sampled during a 300-ns molecular dynamics simulation.

**Figure 13:**
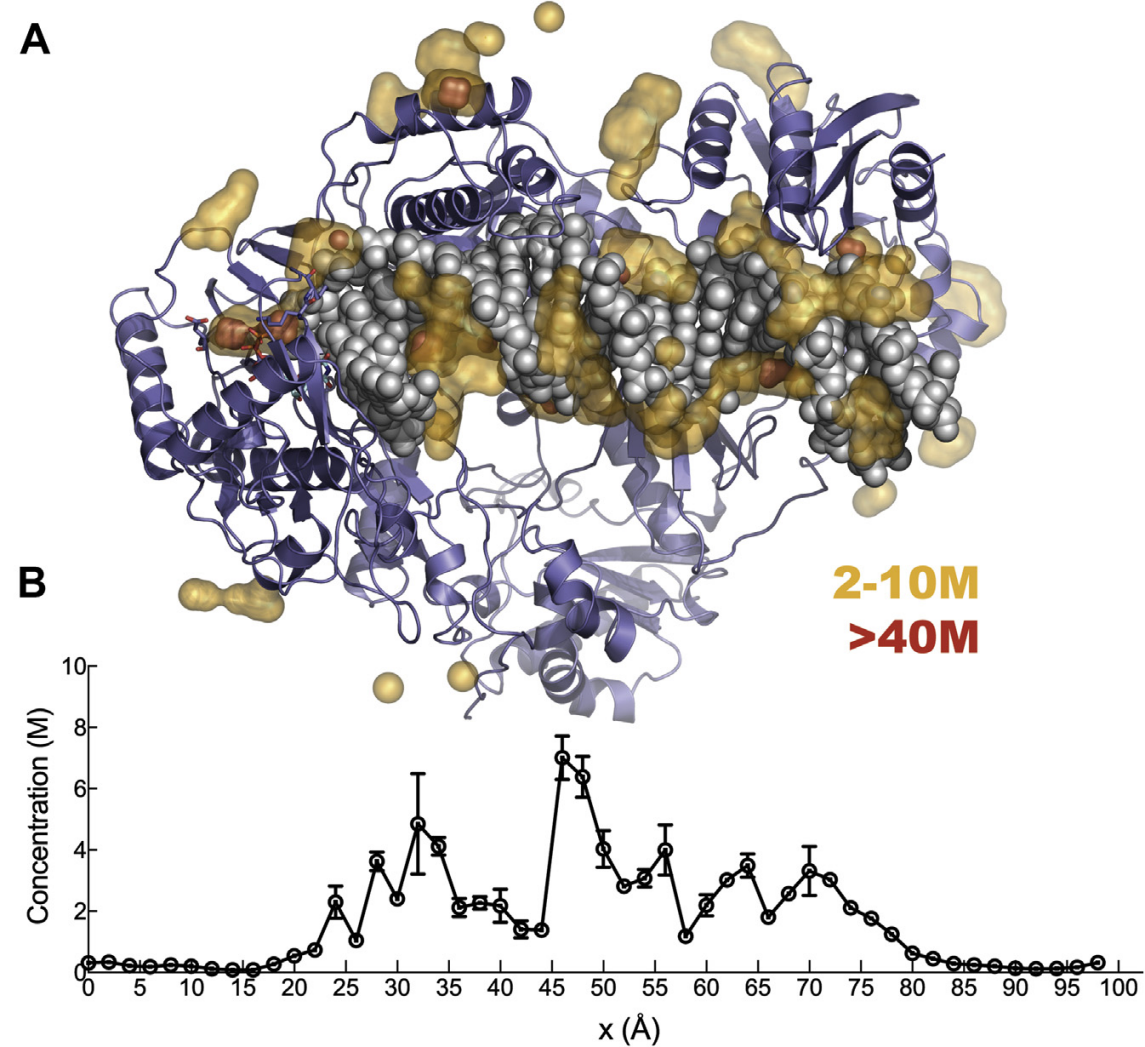
***A*, overview of the Mg**^**2+**^**ion occupancy around the HIV-RT.** Local concentrations shown in heatmap, sticks represent the incoming nucleotide and two aspartic acid side chains. Protein is shown in *cartoon representation* and DNA in *sphere*. *B*, Mg^2+^ ion density profile along the long DNA axis. The two images aligned for a clear view of the positions of the localized ions.

Figure 12 shows the distribution of Mg^2+^ ions around the enzyme–DNA–Mg.dNTP ternary complex, where each dot represents a Mg^2+^ position observed during the simulation. To show the correlation of the positions sampled by Mg^2+^ ions we combine snapshots taken every 1 ns to give a visual image, reflecting the probability density distribution. The most obvious conclusion of this analysis is that there is a dense cloud of Mg^2+^ counterions surrounding exposed DNA and exposed charges on the surface of the protein. To further quantify the cation distribution, we plot the constant density regions as a heatmap (Fig. 13*A*). Details of how the ion densities were computed can be found in the Experimental Procedures section. Here, the yellow regions show local Mg^2+^ concentrations in the range of 2 to 10 M, whereas the regions colored in red represent concentrations greater than or equal to 40 M. Simulations suggest that a cloud of Mg^2+^ counter-ions surrounds the exposed DNA, as described by Manning theory (26), giving rise to an average local concentration of [Mg^2+^] ≈ 2.8 M near the DNA with an average number of bound Mg^2+^ ions of $N_{Mg^{2+}}\approx \;9.8$. Note that we performed the MD simulation using a bulk solution concentration of 30 mM to have a statistically significant number of ions in the simulation box. However, the excess cations surrounding the duplex is expected to be relatively insensitive to the bulk solution concentration (27).

In Figure 13*B*, we project the local concentration of Mg^2+^ ions along the axis of the DNA helix. To compute the local cation concentration around DNA, we create a cylindrical shell of 15 Å radius covering the DNA. Of interest, the average concentration of Mg^2+^ ions around the DNA is not uniform along the DNA axis (Fig. 13*B*); it is highest at the center of the DNA where the DNA is most exposed to the solvent. Regions of lower concentration at 35 to 45 Å and 55 to 65 Å coincide well with the thumb-site and RNase H domains, respectively. The positively charged residues at these domains create a depletion zone for free Mg^2+^ ions. The important conclusion from these observations is that a significant density of Mg^2+^ counter-ions surrounds the DNA. These cations likely impact the binding of DNA as well as the translocation of DNA during processive polymerization. The counterion atmosphere is not seen in the crystal structures because they bind diffusively, but they are important, nonetheless.

**Figure 14:**
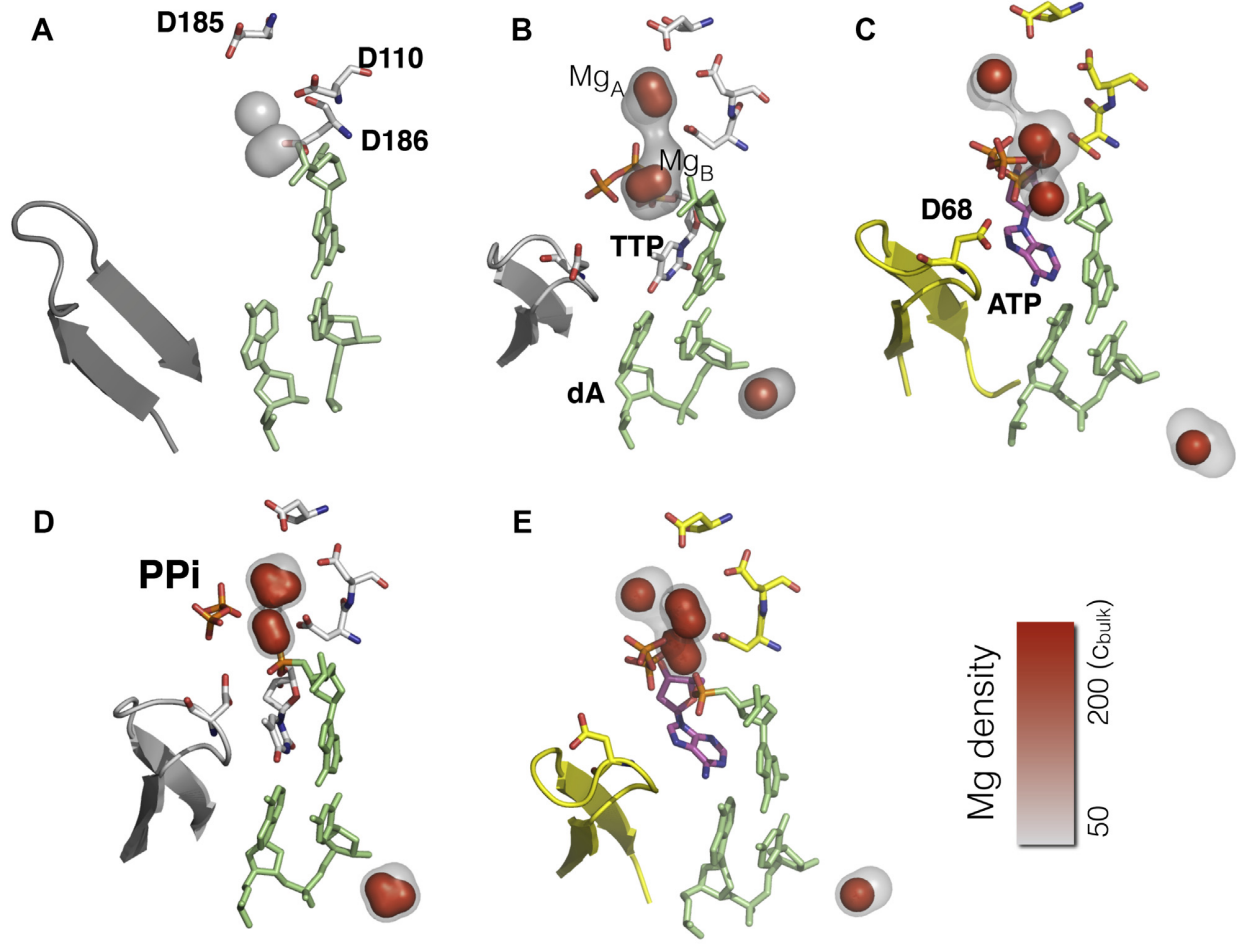
**Mg^2+^ ion density profiles for different steps of polymerase reaction.** Local ion densities are computed by dividing the space into cubic grids. Only high-density regions are shown for clarity. The 3d densities are shown with surface representation; *gray* to *red* color represents regions with ion density from *c* = 50*c*_bulk_ to *c* = 200*c*_bulk_. *A*, corresponds to the ion density when the enzyme is in the open state, *B*, when the substrate is a matching nucleotide TTP:dA, *C*, for the mismatching nucleotide with the template ATP:dA. *D*, after the chemistry step and before the PPi group dissociation from the active site for the matching nucleotide. *E*, the Mg ion density in the mismatched nucleotide after the chemistry step.

In addition to the diffusively bound counterions, MD simulations identify the specifically bound Mg^2+^ ions in agreement with crystal structures as discussed in the previous section. The comparison of metal ion–binding sites with crystal structures allows us to benchmark simulation results and to further extrapolate the metal ion coordination to functional states where crystal structures are not readily available. Figure 14 summarizes our results for Mg^2+^ ion coordination along the polymerase reaction pathway for matching and mismatching dNTP bound states.

In the open state and in the absence of Mg.dNTP, two binding sites in the vicinity of D110 and D185 were observed (Fig. 14*A*) with local concentrations of $c\;\approx \;50c_{bulk},\;$ giving rise to a $K_{d}=\left(\frac{c}{c_{bulk}}\;\frac{1}{mol/L}\right)\;\approx \;20mM.$ The weak Mg^2+^ binding to the *open* state is consistent with available crystal structures reporting the lack of observable Mg^2+^ ions in the vicinity of D110-D185 in the absence of nucleotide (28). After Mg.dNTP binding and the subsequent conformational change to the closed state, MD simulations show an increase in Mg^2+^ binding occupancy between the two aspartate residues (D110 and D185), providing a clear evidence for a dynamically changing electrostatic environment during conformational change following Mg.dNTP binding. The equilibrium positions of the nucleotide and Mg^2+^ ion coincide well with crystal structures (Fig. 11). During the simulation Mg^2+^ cations stay hydrated owing to the reported slow exchange of water from the first solvation shell of Mg^2+^ (29, 30, 31). However, Mg_A_ appears to be chelated to D110 and D185 residues in the crystal structures (Fig. 11 and Table 5), but the nontetrahedral geometry discussed above could be due to hydration of Mg_A_ and errors in structure refinement. The question of whether the Mg^2+^ remains hydrated or forms a direct (chelation) interaction with the carboxylate oxygens is a complicated topic that will be addressed in a subsequent paper.

Next, we studied the Mg^2+^ coordination in a mismatched ternary complex in the closed state. The Mg^2+^-binding sites in a mismatched complex (Mg.dATP with template dA) are shown in Figure 14*C* for comparison. Unlike the matched nucleotide, the equilibrium position of the mismatch leads to misalignment of the incoming base, consistent with our previous observations (32). Interestingly, the misalignment of the mismatch rotates the phosphate group to a bridging position between two negative charges from D68 and the terminal strand of the growing DNA. The increase in the exposed charge density in that region resulted in the formation of a *third* metal ion site owing to counterion condensation. The presence of a third metal ion has been reported in repair enzymes (13, 15). Our simulations suggest the possibility of a third metal ion in a high-fidelity enzyme when mismatched nucleotides are bound, but the location of the third metal ion differs from that reported previously. Care must be exercised in the interpretation of the result. Simulations suggest that the third metal ion appears only when there is a mismatch/improper alignment of the base. The dwell time of the third ion is about 8 ± 2 ns. Given a lifetime of 10 ns and diffusion-limited binding (1 × 10^9^ M^−1^ s^−1^), we estimated a K_d_ ≅ 100 mM for the site of the third metal ion. Hence, the rapid exchange of the third metal ion would make its direct observation challenging by crystallography and it is unlikely to be important under physiological conditions ([Mg^2+^] < 1 mM). Also, the weak binding affinity suggests that this site can be occupied by monovalent ions such as K^+^ or Na^+^ as they are more abundant under physiological conditions.

We also examined the Mg^2+^ ion coordination after the chemistry step, where the phosphodiester bond has just been formed and the by-product PPi is still bound to the complex. Similar to the previous analysis we compare the matched with a mismatched base pair (Fig. 14, *D*–*E*). The matched nucleotide and PPi created two Mg^2+^-binding sites. The binding positions are similar to the ones observed before the chemistry (Fig. 14*B*). In contrast, a mismatched base accumulates three Mg^2+^ ions in the product complex (Fig. 14*E*), in parallel to the mismatch before the chemistry (Fig. 14*C*). Note that all simulations discussed in this section are independent runs started from random Mg^2+^ ion positions. One implication of our finding is that the third metal ion provides extra electrostatic stabilization to the negatively charged PPi at the active site for the mismatch. This is consistent with our observation that PPi release is slower after the incorporation of a mismatched nucleotide (20). Further simulations are underway to study the dissociation rate of the PPi group from the active site with a mismatch to complement previously published simulation studies of the release of PPi after incorporation of a correct base pair (11).

**Figure 15:**
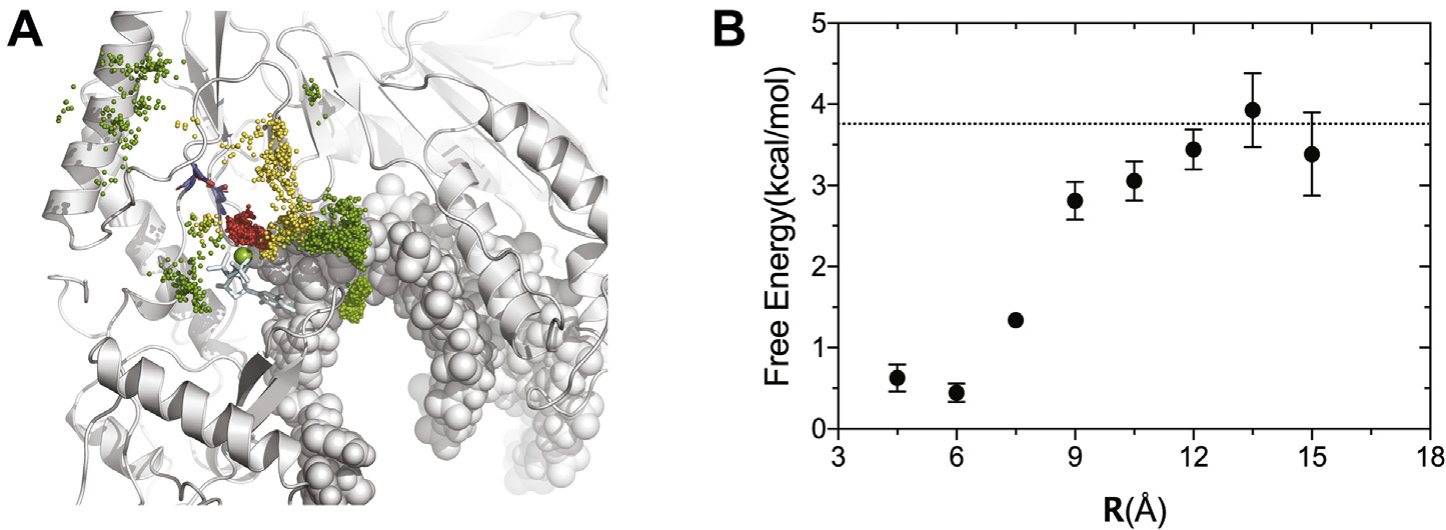
**The reaction coordinate and free energy of Mg**_**A**_**association/dissociation.***A*, we define the reaction coordinate as the closest distance from any free Mg^2+^ ion to the aspartic acid pocket formed by D110 and D185. Different colored spheres represent the spatial distribution of Mg^2+^ ions at different milestones of the pathway for Mg^2+^ dissociation: *green* is for distance of *R* = 15 Å, *yellow* 10 Å, and *red* 5 Å, respectively. As the distance increases, the distribution of Mg^2+^ ions becomes more disperse. *B*, the free energy change along the reaction coordinate is shown with error bars. The *dashed line* represents the free energy difference estimated from the experimentally measured apparent *K*_*d*_ =3.7 mM for binding to the closed state of the enzyme in the presence of nucleotide.

To study the kinetics and thermodynamics of the Mg^2+^ coordination to the Mg_A_ site where we measure the exchange experimentally, we used the milestoning method (33). Unlike the previous cases, where spontaneous association/dissociation of Mg^2+^ ions to the negatively charged surfaces on the enzyme is directly observable on the MD simulation timescale, owing to the relatively higher binding affinity of Mg_A_ the dissociation of magnesium ion is not within the reach of direct MD simulations. The milestoning method allowed us to overcome the timescale problem and to study the thermodynamics and kinetics of the exchange of Mg^2+^ ion from the catalytic site. Rather than following a unique Mg^2+^ ion among the pool of many in the simulation box, we monitored the *closest* Mg^2+^ ion to the unoccupied Mg_A_-binding site, as illustrated in Figure 15*A*. This way the effect of the finite concentration is taken into account and we treat the Mg^2+^ ions as indistinguishable. Details of our approach is in the Experimental Procedures section. The reaction coordinate and the free energy change as a function of the closest Mg^2+^ ion distance is shown in Figure 15*B*. The bound state is about 3.5 kcal/mol more stable relative to a vacant active site. The dwell time of a bound Mg^2+^ at the site on the other hand is found to be 330 ns. The apparent K_d_ of 3.7 mM measured from our experiments (Fig. 8*A*) provides an estimate of 3.2 kcal/mol. This agreement provides an important check for the reliability of our methodology. This also explains, for instance, that EDTA can instantly stop the polymerization reaction in our rapid-quench experiments (34). If the Mg^2+^ dissociated more slowly, there would be a lag in stopping the reaction owing to the slow Mg^2+^ dissociation.

**Figure 16:**
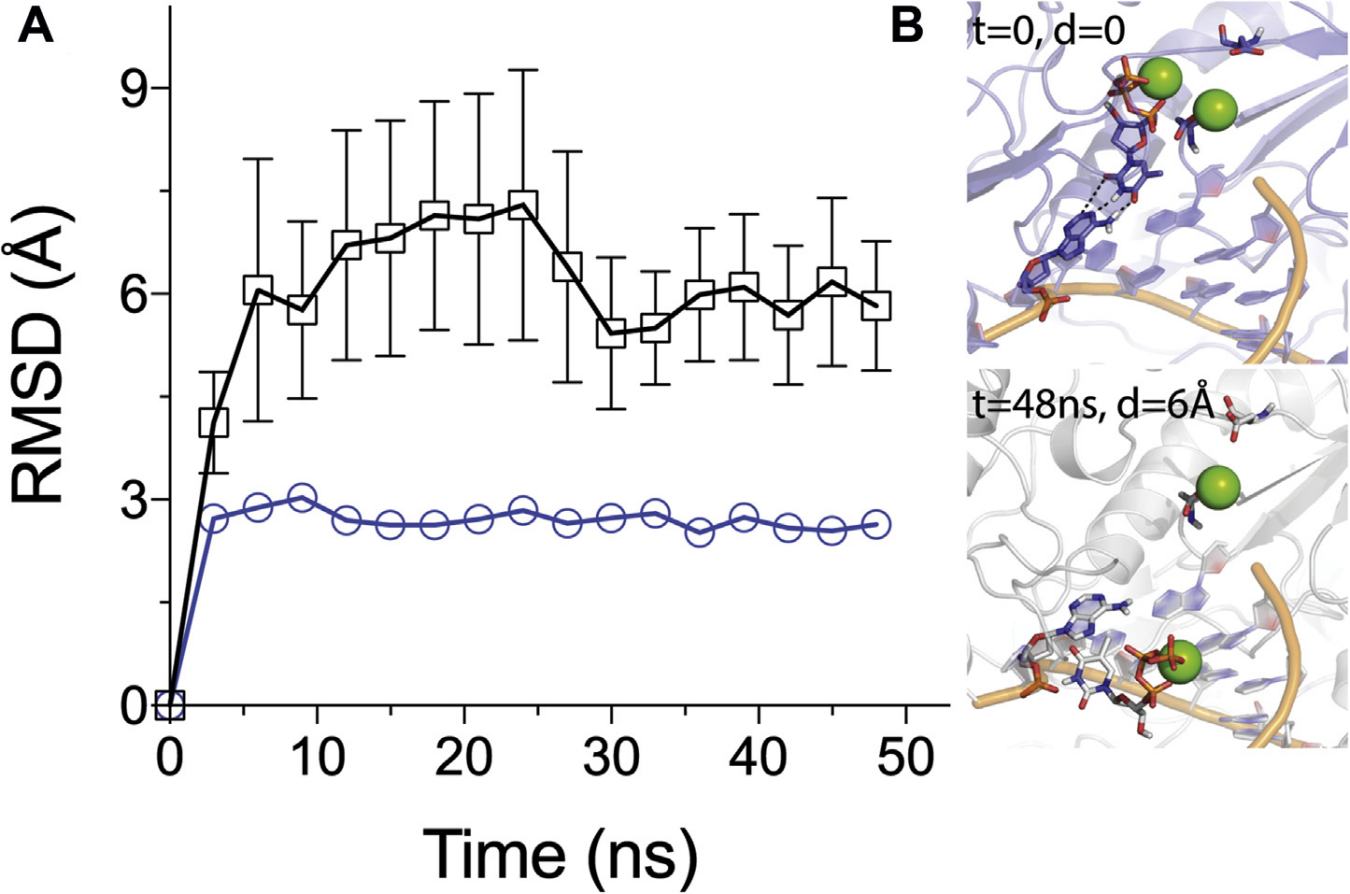
**Time evolution of the root-mean-square deviation (RMSD) of dNTP from its bound state in two possible Mg**_**A**_**binding modes.***A*, If Mg_A_ is chelated to active site residues (D110–D185), electrostatic repulsion causes the MgdNTP to dissociate over time (*black squares*). Alternatively, if the catalytic site is weakly occupied by a hexahydrated Mg_A_, the MgdNTP remains close (*blue circles*) as hexahydrated Mg_A_ is displaced. Averages and error bars are computed from 10 independent simulations. *B*, representative structures from the beginning and the end of a simulation with a chelated Mg_A_ showing dissociation of MgdNTP.

Although there is no crystallographic evidence for the tight binding of Mg_A_ to the open enzyme state, we used MD simulation to consider the consequences of the formation of an ASP-Mg complex in the open state. Through simulations we observe that the tight binding of Mg^2+^ to the aspartic acid residues prevents the proper alignment of Mg.dNTP (Fig. 16). In the absence of Mg_A_, the free carboxylate ligands help to stabilize the Mg.dNTP. In contrast, tightly bound (chelated) magnesium at the site results in misalignment of Mg.dNTP owing to the electrostatic repulsion of the two Mg^2+^ ions. These results suggest that the weak binding of Mg^2+^ (hydrated) can easily be displaced by competition with the incoming Mg.dNTP. Therefore, at physiological Mg^2+^ concentrations, Mg.dNTP is able to bind to the open state of the enzyme without interference by Mg_A_. Analysis of whether Mg_A_ is hydrated or chelated by direct interaction with the carboxylate ligands is a complex problem that must be addressed by more extensive calculations.

## Discussion

It has been more than 2 decades since the general two-metal-ion mechanism for phosphoryl-transfer reactions was proposed (1). Although the metal ions are directly observable in many crystal structures, biochemical experiments are required to establish the kinetic and thermodynamic basis for the roles of the two metal ions in specificity and catalysis. The Mg.dNTP complex is stable thermodynamically (*K*_*d*_ = 28 μM), but the metal ions exchange rapidly with a half-life of 0.1 ms (35), which complicates experimental analysis of the role of the second metal ion. Experiments using the physiologically relevant Mg^2+^ concentrations are needed to examine the two-metal-ion mechanism based upon measurements of the kinetics of binding and catalysis. By accurate calculation of the concentrations of free Mg^2+^ and Mg.dNTP, we kinetically and thermodynamically resolved the participation of the two Mg^2+^ ions. The much weaker binding of the catalytic metal ion affords resolution of the roles of the two metal ions by titrations of activity *versus* free Mg^2+^. In our experiments all Mg^2+^ concentrations were above those needed to saturate the Mg.dNTP complex (≥0.25 mM) and provide Mg^2+^ sufficient to populate the counter-ion cloud around the exposed DNA. We show kinetically that the Mg.dNTP complex binds to induce the conformational change from the open to the closed state and that the catalytic Mg^2+^ binds after the conformational change. Because the Mg–nucleotide complex is the natural substrate for many enzymes, the studies performed here also provide a more physiologically relevant assessment of the role of free Mg^2+^ ions *in vivo*, which may be applicable to a large number of enzymes.

It has been shown that the kinetic partitioning between the reverse conformational transition leading to release of bound nucleotide (limited by *k*_*−2*_) *versus* the forward reaction (*k*_*3*_) is a critical factor defining nucleotide specificity (*k*_cat_/*K*_*m*_). Our results show that nucleotide specificity varies as a function of the free Mg^2+^ concentration owing to weak binding of the catalytic Mg^2+^ (*K*_*d,app*_ of 3.7 mM). High specificity in DNA polymerases is achieved by an induced-fit mechanism in which the enzyme closes rapidly in recognition of a correctly aligned substrate that is bound tightly as the enzyme aligns catalytic residues to facilitate the subsequent binding of the catalytic Mg^2+^ to simulate the chemical reaction. A mismatched nucleotide fails to stabilize the closed state or to align catalytic residues to promote catalysis, so the mismatch is released rather than react (18, 32). The catalytic Mg^2+^ contributes to high fidelity by affecting the kinetic partitioning between going forward for the chemistry *versus* the reverse reaction to release the substrate. In a subsequent article, we will show that fidelity and processivity increase with increasing Mg^2+^ concentration.

Our results resolve the controversy over the variable occupancy of the catalytic metal ion site (36) and support the theory that the reaction is catalyzed by the two-metal-ion mechanism (37). Because the second metal ion binds so weakly, it is not always observed in crystal structures at limited concentrations of Mg^2+^. The results in our study showed that Mg.dTTP binds tightly to HIV-RT in the closed state (*K*_*d,net*_ = 0.5 μM), whereas the catalytic Mg^2+^ binds to HIV-RT relatively weakly (*K*_*d,app*_ is 3.7 mM). The binding of Mg^2+^ to nucleotide (28 μM) is also much tighter (130-fold) than the binding of Mg^2+^ to the active site of the closed E.DNA.MgdNTP complex. Our conclusion that two Mg^2+^ ions bind with different affinities and in sequential order to the active site of HIV-RT during the catalytic cycle is supported by available structural data. Our molecular simulations also confirm the existence of these two binding sites. Their relative binding affinities computed from simulations are consistent with our kinetic data both in the open and closed states. High-resolution x-ray crystal structures of HIV-RT have been obtained at various stages of the reaction with enzyme in complex with nucleotide analog inhibitors (38). These structures show that HIV-RT can bind up to four functionally relevant Mg^2+^ ions, two at the HIV-RT polymerase domain and two at the RNase H domain (36, 39). There is no evidence to support the participation of a third metal ion in cognate nucleotide incorporation. However, simulations explored a weak third metal ion–binding pocket in mismatch incorporation when the only cation in solution is Mg^2+^, but the third metal is far from the catalytic center, and we suggest that monovalent cations abundant in physiological conditions would occupy this site.

We propose that the weak binding of the catalytic Mg^2+^ is an important component contributing to high fidelity. The binding of the second Mg^2+^ is required for catalysis as shown directly by our measurements, supporting the proposals first put forth in postulates of the two-metal-ion mechanism (1). In addition, the second Mg^2+^ stabilizes the closed state by reducing the rate at which the enzyme opens to release the Mg.dNTP. The relatively low affinity of the second Mg^2+^ relative to the physiological concentration may provide an important contribution toward fidelity. A higher Mg^2+^ binding affinity might otherwise stabilize the binding and lead to the incorporation of a mismatched nucleotide. We are led to a model in which fidelity is largely determined by nucleotide binding to the open enzyme state and the conformational change to align the substrate at the active site, followed by weak and presumably transient binding of the Mg^2+^ to stimulate catalysis. Nucleotide selectivity is based on partitioning of the closed state to go forward rather than reverse to release the bound Mg.dNTP, and the binding of the catalytic Mg^2+^ to an aligned correct substrate increases the rate of the chemical step to drive the kinetic partitioning forward.

The role of the catalytic Mg^2+^ binding is supported by several experiments. The temperature-dependent stopped-flow experiment was repeated with three free Mg^2+^ concentrations (0.25, 1, and 10 mM) showing no obvious effect of free Mg^2+^ on the rate of forward conformational change (*k*_*2*_). In addition, the global fitting of four experiments also showed that the free Mg^2+^ concentration has a minimal effect on the ground-state binding (*K*_*1*_). These results demonstrate that catalytic Mg^2+^ binds after the enzyme closes. To further test this hypothesis, double mixing experiment was also performed in which Mg.dTTP was first mixed with the enzyme–DNA complex in the presence of very low free Mg^2+^ to allow the enzyme closing (Fig. 9*D*). After a large excess of free Mg^2+^ was added at the second mixing, the chemistry occurred immediately. These results further demonstrate that the catalytic Mg^2+^ is not required for the nucleotide-induced forward conformational change but is required for catalysis.

Another approach that has been used toward dissecting the roles of the two metal ions is based on the use of the exchange-inert Rh-dNTP complex that can be purified and then mixed with the enzyme to examine the kinetics of the conformational change in the absence of Mg^2+^ (40, 41). These studies were performed before accurate measurements of the rates of the conformational change and rely on the assumption that Rh.dNTP accurately mimics Mg.dNTP. Therefore, it was necessary to make direct measurement of the effect of free Mg^2+^ concentration on each step in the pathway, including the nucleotide-induced conformational change. As part of this study we show that the Rh.dNTP complex induces a change in structure of the enzyme from the open to the closed state in the absence of excess Mg^2+^, but with altered kinetics. The results from all three experiments are also consistent with the result from the [Rh-dTTP]^2−^ binding experiment, suggesting that nucleotide-bound Mg^2+^ is sufficient for inducing the enzyme closing.

Recently, it has been reported that the third Mg^2+^ is transiently bound during nucleotide incorporation, and the existence of the third Mg^2+^ was proposed to affect PPi release (15). The rates of the PPi release were not accurately defined in our experiments other than to show that PPi release is coincident with the observed rate of polymerization for correct nucleotide incorporation at the free Mg^2+^ concentrations ranging from 0.25 to 10 mM. Our previous studies on the mismatched incorporation using RNA/DNA duplex indicated that the rate of PPi release is slow (∼0.03 s^−1^) and rate limiting (20). Further studies on the mismatched incorporation using DNA/DNA duplex are needed to directly compare the rates of PPi release for correct nucleotide incorporation *versus* mismatched incorporation. If the rate of the PPi release is indeed very slow in mismatched nucleotide incorporation, it would suggest that the nucleotide-bound Mg^2+^ itself is not sufficient for facilitating the PPi release, and the proper alignment in the active site or possibly the third Mg^2+^ is required to facilitate PPi release. Pyrophosphorolysis cannot be detected (<1%) with a mismatched primer/template complex over the time scale of 4 h, suggesting an apparent rate constant of less than 10^−6^ s^−1^. These data suggest that the binding of Mg^2+^ and PPi does not provide sufficient energy to overcome the misalignment of the mismatched primer terminus to reach a catalytically competent state.

Finally, we investigated nucleotide specificity (*k*_cat_/*K*_*m*_) at different Mg^2+^ concentrations. Our results show that the rate of nucleotide incorporation is Mg^2+^ dependent. The *k*_cat_/*K*_*m*_ value for dTTP incorporation decreased approximately 12-fold as the free Mg^2+^ concentration was decreased from 10 to 0.25 mM (Table 2). It is known that the physiological Mg^2+^ concentration varies in different cell types (42, 43). For example, it is reported that the physiological concentration of free Mg^2+^ in human T lymphocytes is around 0.25 mM (44, 45), but around 0.6 mM in mammalian muscle cell (46). Given the weak Mg^2+^ binding affinity, the Mg^2+^-dependent nucleotide incorporation found in our experiment may be one of the mechanisms used to regulate the activities of some enzymes. On the other hand, it is possible that HIV perturbs the intracellular Mg^2+^ concentration to optimize viral replication. We are currently measuring the Mg^2+^ concentration dependence of misincorporation, which is needed to fully assess the role of Mg^2+^ concentration on fidelity.

In conclusion, we have investigated the role of each Mg^2+^ ion in the two-metal-ion mechanism by studying their binding affinities, binding mode (sequential binding or simultaneous binding), and the effects of their binding on each individual step leading to nucleotide incorporation. The studies we have performed here provided insight and detailed information about the general two-metal-ions mechanism that may be applicable to many enzymes.

## Experimental procedures

### Mutagenesis, expression, and purification of MDCC-labeled HIV-RT

HIV-RT protein was expressed, purified, and labeled without resorting to the use of tagged protein as described (18). Briefly, cysteine mutants of the p51 (C280S) and p66 (E36C/C280S) subunits of HIV-RT were separately expressed and then cells were combined to yield a 1:1 ratio of the two subunits, lysed, sonicated, and then the heterodimer was purified. The protein was first purified by using the tandem Q-Sepharose and Bio-Rex70 columns and further purified by using a single-stranded DNA (ssDNA) affinity column. The protein was then labeled with the MDCC (7-diethylamino-3-[*N*-(2-maleimidoethyl) carbamoyl]coumarin) from Sigma-Aldrich. Unreacted MDCC was removed by ion exchange using a Bio-Rex70 column. After purification, the “Coomassie Plus” protein assay was used to estimate the purified protein concentration (ThermoFisher). In addition, an active site titration was performed to determine the active site concentrations of the purified protein (17), which was used in all subsequent experiments.

### Preparation of DNA substrates for kinetic studies

The 25/45 and 25ddA/45-nt DNA substrates were purchased from Integrated DNA Technologies, using the following sequences:

25 nt: 5'-GCCTCGCAGCCGTCCAACCAACTCA-3'

45 nt: 5'-GGACGGCATTGGATCGACGATGAGTTGGTTGGACGGCTGCGAGGC-3'

The oligonucleotides were annealed by heating at 95 °C for 5 min, followed by slow cooling to room temperature. For making the radiolabeled primer, the 25-nt oligonucleotide was labeled at the 5’ end by γ-^32^P ATP (PerkinElmer) using T4 polynucleotide kinase (NEB).

### Quench flow kinetic assays

Rapid chemical-quench-flow experiments were performed by mixing a preformed enzyme–DNA complex (using radiolabeled DNA primer) with various concentrations of incoming nucleotide using a KinTek RQF-3 instrument (KinTek Corp, Austin, TX, USA). The reaction was quenched by the addition of 0.5 M EDTA at varying time points. The products were collected and separated on 15% denaturing PAGE (acrylamide [1:19 bisacrylamide], 7 M urea). The results were then analyzed using ImageQuant 6.0 software (Molecular Dynamics).

### Stopped flow kinetic assays

The stopped-flow measurements were performed by rapidly mixing an enzyme–DNA complex (using MDCC-labeled HIV-RT) with various concentrations of incoming nucleotide. The time dependence of fluorescence change upon nucleotide binding and incorporation was monitored using an AutoSF-120 stopped-flow instrument (KinTek Corp) by exciting the fluorophore at 425 nm and monitoring the fluorescence change at 475 nm using a band-pass filter with a 25-nm bandwidth (Semrock).

### Equilibrium titration assays

Equilibrium titration experiments were performed by titrating a 0.25 μl solution containing a preformed enzyme–_dd_DNA complex (100 nM MDCC-labeled HIV-1 wildtype RT and 150 nM DNA with a dideoxy-terminated primer) with increasing concentrations of the incoming nucleotide. The signal change was monitored continuously using the TMX titration module accessor on the SF-300x stopped-flow instrument (KinTek Corp). Fluorescence was excited at 425 nm and monitored at 475 nm using a band-pass filter with a 25-nm bandwidth (Semrock). The fluorescence signal was corrected for the small dilution during the titration.

### Global fitting of multiple experiments

The kinetic parameters governing each step leading to nucleotide incorporation were obtained by globally fitting four experiments (stopped-flow, chemical-quench, nucleotide off-rate, and PPi release) using the model shown in Figure 1 with *KinTek Explorer* software (KinTek Corp). Fitspace confidence contour analysis was also performed to estimate standard errors (47, 48).

### Free Mg^2+^ concentration calculation

To calculate the free Mg^2+^ concentration in solution, EDTA (500 μM) was used as a buffering system. The main equilibria that affect free Mg^2+^ concentration are the equilibrium constants for Mg^2+^ binding to dNTP, Mg^2+^ binding to EDTA, and the equilibrium for protonation of dNTP (shown in Table 6). Calculation of the free Mg^2+^ concentration from starting total concentrations of Mg^2+^, dNTP, and EDTA and the pH requires an iterative approach to solve simultaneously the equilibria involved. However, we simplified the problem by specifying the desired free Mg^2+^ concentration and total EDTA concentration and then calculating the total concentrations of Mg^2+^ and dNTP that must be added to the solution using a simplified set of equations:

(3) $$\begin{array}{l} Mg\;+\;dNTP\overset{K_{1}}{⇌}Mg.dNTP\\ Mg\;+\;EDTA\overset{K_{2}}{⇌}Mg.EDTA\\ H\;+\;dNTP\overset{K_{3}}{⇌}H.dNTP\\ {\left[Mg\right]}_{0}=\left[Mg\right]\;+\;\left[Mg.dNTP\right]\;+\;\frac{{\left[EDTA\right]}_{0}}{1\;+\;K_{2}/\left[Mg\right]}\\ {\left[dNTP\right]}_{0}=\left[Mg.dNTP\right]\;\cdot \;(1\;+\;\left(K_{1}/\left[Mg\right]\right)\;\left(1\;+\;\left[H\right]/K_{3}\right)) \end{array}$$

**Table 6:** Equilibrium constants used for the calculation of concentrations of free magnesium and Mg.dNTP concentration*s*

Equilibrium	*K*_*a*_ (M^−1^)	*K*_*d*_ (μM)
$Mg^{2+}+ATP^{4-}⇄Mg.ATP^{2-}$	34,800	28.7
$H^{+}+ATP^{4-}⇄H.ATP^{3-}$	1.09 × 10^7^	9.17 × 10^−2^
$H^{+}+H.ATP^{3-}⇄H_{2}.ATP^{2-}$	8500	118
$Mg^{2+}+H.ATP^{3-}⇄Mg.H.ATP^{-}$	542	1845
$Mg^{2+}+EDTA^{4-}⇄Mg.EDTA^{2-}$	4 × 10^8^	0.25 × 10^−2^
$H^{+}+EDTA^{4-}⇄H.EDTA^{3-}$	1.66 × 10^10^	6 × 10^−5^
$H^{+}+H.EDTA^{3-}⇄H_{2}.EDTA^{2-}$	1.58 × 10^6^	0.633

The association and dissociation constants, Ka and Kd, respectively, were obtained from (Storer *et al.*, 1976; Martell *et al.*, 1964) (8, 9).

To confirm the accuracy of our approximation, the reactions were simulated with all equilibria (Table 6) (8, 9) using the *KinTek Explorer* software. In this case, starting total concentrations of Mg^2+^, dNTP, and EDTA were entered and the free Mg^2+^ concentration was directly calculated after the system reached equilibrium. This kinetic approach to reach equilibrium circumvents the typical semirandom search for a mathematical solution and yet still affords a simultaneous solution of the multiple equilibria.

### Calculation of steady-state kinetic parameters

Steady-state kinetic parameters were calculated from the intrinsic rate constants using the following equations according to Figure 1, simplified by the known fast product release (*k*_*4*_ >> *k*_*3*_). The initial ground-state binding was modeled as a rapid equilibrium with *k*_*1*_ = 100 μM^−1^ s^−1^. Estimates of the remaining rate constants were then used to calculate the steady-state kinetic parameters.

(4) $$\begin{array}{l} k_{cat}=\frac{k_{2}k_{3}}{k_{2}\;+\;k_{-2}\;+\;k_{3}}\\ K_{m}=\frac{k_{2}k_{3}\;+\;k_{-1}\left(k_{-2}\;+\;k_{3}\right)}{k_{1}\left(k_{2}\;+\;k_{-2}\;+\;k_{3}\right)}\\ k_{cat}/K_{m}=\frac{k_{1}k_{2}k_{3}}{k_{2}k_{3}\;+\;k_{-1}\left(k_{-2}\;+\;k_{3}\right)} \end{array}$$

### Initial state of MD simulation models

Initial states of the ED-Mg.dNTP ternary complex were based on the open (1j5o) and closed (1rtd) state structures of HIV1-RT from the protein databank (49). Five independent simulation setups were prepared as follows: 1) open-state enzyme with no bound dNTP; 2) closed-state enzyme with matching nucleotide, [Mg.dTTP]^−2^ opposite to a templating base adenine forming dTTP:dA pair; 3) closed-state enzyme with a mismatching nucleotide, [Mg.ATP]^−2^ opposite to templating DNA forming dATP:dA pair; 4) closed-state enzyme after the chemistry step where a matching nucleotide is added to the DNA strand and the pyrophosphate (PPi) group is bound; 5) closed-state enzyme after chemistry step where a mismatching nucleotide added and PPi is bound.

### MD simulation setup

MD simulations were performed using the GROMACS suit of programs (50). Each setup was first energy minimized with the steepest descent method for 5000 steps followed by solvation with explicit water with a minimum of 12 Å thickness from the surface of the complex, giving rise to a simulation box of about 126.6 x 125.2 x 112.7 Å^3^. To neutralize the simulation box and to mimic experimental conditions we added 51 Mg^2+^ and 63 Cl^−^ by randomly replacing water molecules with ions, resulting in 30 ∓ 1 mM free Mg^2+^ in the bulk after equilibration. Water was represented by the SPC/E model (51). Protein, DNA, and Cl^−^ molecules were represented by default Amber03 forcefield parameters (52). For Mg^2+^ ions we used a recently developed parameter (31) that shows better agreement with solution exchange rates. The parameters of dTTP, dATP, and PPi were adopted from Amber forcefield while charges were computed from quantum mechanics as explained in our earlier work (32).

We computed the long-range interactions with a distance cutoff of 12 Å with dispersion correction for van der Waals interactions (53) and Particle Mesh Ewald summation method (54) for electrostatic. Electrostatic interactions were computed with a grid spacing of 1.15, 1.16, and 1.12 Å in directions *x*, *y*, and *z*. Equations of motion were integrated by Leapfrog integrator (55) with a time step of 2 fs. All bonds were constrained using the LINCS (56) algorithm.

Following the energy minimization for each solvated system we conducted a two-step equilibration process: the first equilibration involves finding the volume of the box that gives rise to 1 atm pressure at 310K; the second equilibration allowed water and ions to equilibrate around the complex. In detail, we sampled the conformations for 2 ns from Isothermal Isobaric ensemble (NPT) using the Parrinello–Rahman scheme (57) and the temperature was kept constant by velocity scaling (54). The positions of heavy atoms of the solute were restrained using harmonic potential with a stiffness constant of 1000 kJ/nm^2^. Using the last frame of the simulation as the starting point, we employed a 200-ns-long constant volume and temperature (NVT) simulation for solvent equilibration. In this stage, the stiffness constant of the position restraints was decreased to 50 J/nm^2^ to allow local adjustments in the enzyme–substrate complex. The last frame of the trajectory was used as a starting point for unconstrained MD simulations where we compute our observables. For sampling equilibrium configurations from NVT ensemble we employed a minimum of 300-ns-long simulation in each setup described above. We removed the translational and rotational degrees of the enzyme for every 10 ps and coordinates of atom positions were recorded for each picosecond for data analysis.

### Computing ion density

To study Mg^2+^ distribution around the complex, we divided the simulation box into cubic grids of 1 Å in each direction and computed the average Mg^2+^ ion occupancy at each grid. From the occupancy, local ion concentrations and free Mg^2+^ concentration at the bulk were computed. To estimate bulk concentration, we averaged the concentrations of all grids that are 12 Å or more away from the enzyme surface.

### Computing the kinetics and thermodynamics of Mg_A_

To study the kinetics and thermodynamics of Mg^2+^ coordination to catalytic site we developed a method employing the milestoning approach. In the milestoning approach we partitioned the phase space into milestones. Using trajectory fragments, we estimated the stationary flux between milestones. Details of the milestoning approach employed can be found in Ref (58, 59). From the stationary flux we computed the average mean first passage time, $\langle \tau \rangle =\underset{a}{\sum }\frac{q_{a}t_{a}}{q_{f}}$, where *q*_*a*_ is the stationary flux of milestone *a*, *t*_*a*_ is the average dwell time at *a*, and *q*_*f*_ is the flux to the product state. In addition, the stationary fluxes can be used to extract the free energy of each milestone using $F_{a}=-k_{b}T\;\ln\left(q_{a}t_{a}\right).$

Milestoning in our study requires a reasonable pathway and a reaction coordinate that quantify the progress of the ion migration process. To obtain Mg^2+^ ion binding pathway we created 25 configurations from equilibrium MD simulations obtained from *Setup 2* described above. Initially, all of these configurations had a Mg^2+^ ion in the Mg_A_ site. We moved the ion to a random place in the simulation box to create a vacancy at the catalytic site. After 100 ps of solvent equilibration of the entire system we monitored the diffusion of all remote Mg^2+^ ions by computing the closest magnesium ion to the carboxylic acids atoms of the pocket created by D110 and D185. Figure 15*A* shows representative milestones at distance values of 15, 10, and 5 ± 0.25 Å, respectively. We employed about 100 ns sampling of ion conformations at various initial states, totaling 2.5 ms of simulation time. These trajectories later used to compute the transition kernel, *K*_ab_, where *a* and *b* correspond to neighboring milestone indices. The milestones are distributed between the initial to the end state in 1-Å intervals. We ensured the convergence of the transition probabilities. To study the association rate, we assigned absorbing boundary conditions to the two Mg-bound states, defined by *R* = 4 Å (product) and a reflecting boundary condition to *R* = *R*_bulk_ (reactant). Here, *R*_bulk_ is defined as the average distance between two Mg^2+^ if they were uniformly distributed in solution. *R*_bulk_ is ∼16 Å in 30 mM free Mg^2+^ concentration. As it is known that the dNTP-bound Mg is already at the active site, this distance is the average distance where one can find another Mg^2+^ ion. We used the association pathway that is accessible to brute force MD to compute the kinetics of the dissociation process. For dissociation rate, the milestoning equations are solved with the boundary conditions reversed.

## Data availability

KinTek Explorer mechanism files used to fit data and refined HIV-RT structure files used for MD simulations are available upon request.

## Conflict of interest

K. A. J. is the President of KinTek Corp, which provided the AutoSF-120 stopped-flow, RQF-3 rapid-quench-flow, and KinTek Explorer software used in this study.

## References

[1] Steitz T.A., Steitz J.A. (1993). A general two-metal-ion mechanism for catalytic RNA. Proc. Natl. Acad. Sci. U. S. A..

[2] Steitz T.A. (1999). DNA polymerases: structural diversity and common mechanisms. J. Biol. Chem..

[3] Yang W. (2008). An equivalent metal ion in one- and two-metal-ion catalysis. Nat. Struct. Mol. Biol..

[4] Adams J.A., Taylor S.S. (1993). Divalent metal ions influence catalysis and active-site accessibility in the cAMP-dependent protein kinase. Protein Sci..

[5] Tesmer J.J., Sunahara R.K., Johnson R.A., Gosselin G., Gilman A.G., Sprang S.R. (1999). Two-metal-Ion catalysis in adenylyl cyclase. Science.

[6] Yang L.J., Arora K., Beard W.A., Wilson S.H., Schlick T. (2004). Critical role of magnesium ions in DNA polymerase beta's closing and active site assembly. J. Am. Chem. Soc..

[7] Fenstermacher K.J., DeStefano J.J. (2011). Mechanism of HIV reverse transcriptase inhibition by zinc: formation of a highly stable enzyme-(primer-template) complex with profoundly diminished catalytic activity. J. Biol. Chem..

[8] Martell L. (1964). Stability Constants of Metal-Ion Complex.

[9] Storer A.C., Cornish-Bowden A. (1976). Concentration of MgATP2- and other ions in solution. Calculation of the true concentrations of species present in mixtures of associating ions. Biochem. J..

[10] Freudenthal B.D., Beard W.A., Shock D.D., Wilson S.H. (2013). Observing a DNA polymerase choose right from wrong. Cell.

[11] Atis M., Johnson K.A., Elber R. (2017). Pyrophosphate release in the protein HIV reverse transcriptase. J. Phys. Chem. B..

[12] Nakamura T., Zhao Y., Yamagata Y., Hua Y.J., Yang W. (2012). Watching DNA polymerase eta make a phosphodiester bond. Nature.

[13] Yang W., Weng P.J., Gao Y. (2016). A new paradigm of DNA synthesis: three-metal-ion catalysis. Cell Biosci..

[14] Stevens D.R., Hammes-Schiffer S. (2018). Exploring the role of the third active site metal ion in DNA polymerase eta with QM/MM free energy simulations. J. Am. Chem. Soc..

[15] Tsai M.D. (2019). Catalytic mechanism of DNA polymerases-Two metal ions or three?. Protein Sci..

[16] Kellinger M.W., Johnson K.A. (2011). Role of induced fit in limiting discrimination against AZT by HIV reverse transcriptase. Biochemistry.

[17] Tsai Y.C., Johnson K.A. (2006). A new paradigm for DNA polymerase specificity. Biochemistry.

[18] Kellinger M.W., Johnson K.A. (2010). Nucleotide-dependent conformational change governs specificity and analog discrimination by HIV reverse transcriptase. Proc. Natl. Acad. Sci. U. S. A..

[19] Johnson K.A. (2019). Kinetic Analysis for the New Enzymology: Using Computer Simulation to Learn Kinetics and Solve Mechanisms.

[20] Li A., Gong S.Z., Johnson K.A. (2016). Rate-limiting pyrophosphate release by HIV reverse transcriptase improves fidelity. J. Biol. Chem..

[21] Huang H.F., Chopra R., Verdine G.L., Harrison S.C. (1998). Structure of a covalently trapped catalytic complex of HIV-I reverse transcriptase: implications for drug resistance. Science.

[22] Hanes J.W., Johnson K.A. (2008). Real-time measurement of pyrophosphate release kinetics. Anal. Biochem..

[23] Hanes J.W., Johnson K.A. (2007). A novel mechanism of selectivity against AZT by the human mitochondrial DNA polymerase. Nucleic Acids Res.

[24] Brune M., Hunter J.L., Howell S.A., Martin S.R., Hazlett T.L., Corrie J.E., Webb M.R. (1998). Mechanism of inorganic phosphate interaction with phosphate binding protein from Escherichia coli. Biochemistry.

[25] Noat G., Ricard J., Borel M., Got C. (1970). Kinetic study of yeast hexokinase. Inhibition of the reaction by magnesium and ATP. Eur. J. Biochem..

[26] Manning G.S. (1984). Limiting laws and counterion condensation in poly-electrolyte solutions 8. Mixtures of counterions, specific selectivity, and valence selectivity. J. Phys. Chem..

[27] Kirmizialtin S., Silalahi A.R.J., Elber R., Fenley M.O. (2012). The ionic atmosphere around A-RNA: Poisson-Boltzmann and molecular dynamics simulations. Biophys. J..

[28] Sarafianos S.G., Das K., Clark A.D., Ding J., Boyer P.L., Hughes S.H., Arnold E. (1999). Lamivudine (3TC) resistance in HIV-1 reverse transcriptase involves steric hindrance with beta-branched amino acids. Proc. Natl. Acad. Sci. U. S. A..

[29] Bleuzen A., Pittet P.A., Helm L., Merbach A.E. (1997). Water exchange on magnesium(II) in aqueous solution: a variable temperature and pressure O-17 NMR study. Magn. Reson. Chem..

[30] Lee Y., Thirumalai D., Hyeon C. (2017). Ultrasensitivity of water exchange kinetics to the size of metal ion. J. Am. Chem. Soc..

[31] Allner O., Nilsson L., Villa A. (2012). Magnesium ion-water coordination and exchange in biomolecular simulations. J. Chem. Theor. Comput..

[32] Kirmizialtin S., Nguyen V., Johnson K.A., Elber R. (2012). How conformational dynamics of DNA polymerase select correct substrates: experiments and simulations. Structure.

[33] Faradjian A.K., Elber R. (2004). Computing time scales from reaction coordinates by milestoning. J. Chem. Phys..

[34] Patel S.S., Wong I., Johnson K.A. (1991). Pre-steady-state kinetic-analysis of processive DNA-replication including complete characterization of an exonuclease-deficient mutant. Biochemistry.

[35] Pecoraro V.L., Hermes J.D., Cleland W.W. (1984). Stability constants of Mg2+ and Cd2+ complexes of adenine nucleotides and thionucleotides and rate constants for formation and dissociation of MgATP and MgADP. Biochemistry.

[36] Cowan J.A., Ohyama T., Howard K., Rausch J.W., Cowan S.M., Le Grice S.F. (2000). Metal-ion stoichiometry of the HIV-1 RT ribonuclease H domain: evidence for two mutually exclusive sites leads to new mechanistic insights on metal-mediated hydrolysis in nucleic acid biochemistry. J. Biol. Inorg. Chem..

[37] Klumpp K., Hang J.Q., Rajendran S., Yang Y., Derosier A., Wong Kai In P., Overton H., Parkes K.E., Cammack N., Martin J.A. (2003). Two-metal ion mechanism of RNA cleavage by HIV RNase H and mechanism-based design of selective HIV RNase H inhibitors. Nucleic Acids Res..

[38] Sarafianos S.G., Clark A.D., Das K., Tuske S., Birktoft J.J., Ilankumaran P., Ramesha A.R., Sayer J.M., Jerina D.M., Boyer P.L., Hughes S.H., Arnold E. (2002). Structures of HIV-1 reverse transcriptase with pre- and post-translocation AZTMP-terminated DNA. EMBO J..

[39] Cristofaro J.V., Rausch J.W., Le Grice S.F., DeStefano J.J. (2002). Mutations in the ribonuclease H active site of HIV-RT reveal a role for this site in stabilizing enzyme-primer-template binding. Biochemistry.

[40] Bakhtina M., Lee S., Wang Y., Dunlap C., Lamarche B., Tsai M.D. (2005). Use of viscogens, dNTPalphaS, and rhodium(III) as probes in stopped-flow experiments to obtain new evidence for the mechanism of catalysis by DNA polymerase beta. Biochemistry.

[41] Lee H.R., Wang M., Konigsberg W. (2009). The reopening rate of the fingers domain is a determinant of base selectivity for RB69 DNA polymerase. Biochemistry.

[42] Valberg L.S., Holt J.M., Paulson E., Szivek J. (1965). Spectrochemical analysis of sodium, potassium, calcium, magnesium, copper, and zinc in normal human erythrocytes. J. Clin. Invest..

[43] Walser M. (1967). Magnesium metabolism. Ergeb. Physiol..

[44] Delva P., Pastori C., Degan M., Montesi G., Lechi A. (1998). Intralymphocyte free magnesium and plasma triglycerides. Life Sci..

[45] Delva P., Pastori C., Degan M., Montesi G., Lechi A. (2004). Catecholamine-induced regulation *in vitro* and ex vivo of intralymphocyte ionized magnesium. J. Membr. Biol..

[46] Tashiro M., Konishi M. (1997). Basal intracellular free Mg2+ concentration in smooth muscle cells of Guinea pig tenia cecum: intracellular calibration of the fluorescent indicator furaptra. Biophys. J..

[47] Johnson K.A., Simpson Z.B., Blom T. (2009). FitSpace Explorer: an algorithm to evaluate multidimensional parameter space in fitting kinetic data. Anal. Biochem..

[48] Johnson K.A., Simpson Z.B., Blom T. (2009). Global Kinetic Explorer: a new computer program for dynamic simulation and fitting of kinetic data. Anal. Biochem..

[49] Berman H.M., Westbrook J., Feng Z., Gilliland G., Bhat T.N., Weissig H., Shindyalov I.N., Bourne P.E. (2000). The protein data bank. Nucleic Acids Res..

[50] Mark James Abraham T.M., Schulz R., Páll S., Smith J.C., Hess B., Lindahl E. (2015). GROMACS: high performance molecular simulations through multi-level parallelism from laptops to supercomputers. SoftwareX.

[51] Berendsen H.J.C., Grigera J.R., Straatsma T.P. (1987). The missing term in effective pair potentials. J. Phys. Chem..

[52] Duan Y., Wu C., Chowdhury S., Lee M.C., Xiong G.M., Zhang W., Yang R., Cieplak P., Luo R., Lee T., Caldwell J., Wang J.M., Kollman P. (2003). A point-charge force field for molecular mechanics simulations of proteins based on condensed-phase quantum mechanical calculations. J. Comput. Chem..

[53] Wennberg C.L., Murtola T., Hess B., Lindahl E. (2013). Lennard-Jones lattice summation in bilayer simulations has critical effects on surface tension and lipid properties. J. Chem. Theor. Comput..

[54] Bussi G., Donadio D., Parrinello M. (2007). Canonical sampling through velocity-rescaling. J. Chem. Phys..

[55] Van Gunsteren W.F., Berendsen H.J.C. (1988). A leap-frog algorithm for stochastic dynamics. Mol. Simulat..

[56] Hess B. (2008). P-LINCS: a parallel linear constraint solver for molecular simulation. J. Chem. Theor. Comput..

[57] Parrinello M., Rahman A. (1981). Polymorphic transitions in single crystals: a new molecular dynamics method. J. Appl. Phys..

[58] Kirmizialtin S., Elber R. (2011). Revisiting and computing reaction coordinates with directional milestoning. J. Phys. Chem. A..

[59] West A.M.A., Elber R., Shalloway D. (2007). Extending molecular dynamics time scales with milestoning: example of complex kinetics in a solvated peptide. J. Chem. Phys..

